# Targeting hepatic oxidative stress rescues bone loss in liver fibrosis

**DOI:** 10.1016/j.molmet.2022.101599

**Published:** 2022-09-13

**Authors:** Soichiro Sonoda, Sara Murata, Haruyoshi Yamaza, Ratih Yuniartha, Junko Fujiyoshi, Koichiro Yoshimaru, Toshiharu Matsuura, Yoshinao Oda, Shouichi Ohga, Tasturo Tajiri, Tomoaki Taguchi, Takayoshi Yamaza

**Affiliations:** 1 Department of Molecular Cell Biology and Oral Anatomy, Kyushu University Graduate School of Dental Science, Fukuoka, Japan; 2 Department of Pediatric Dentistry, Kyushu University Graduate School of Dental Science, Fukuoka, Japan; 3 Department of Anatomy, Faculty of Medicine, Public Health, and Nursing, Universitas Gadjah Mada, Yogyakarta, Indonesia; 4 Department of Pediatrics, Kyushu University Graduate School of Medical Sciences, Fukuoka, Japan; 5 Department of Pediatric Surgery, Kyushu University Graduate School of Medical Sciences, Fukuoka, Japan; 6 Department of Anatomic Pathology, Kyushu University Graduate School of Medical Sciences, Fukuoka, Japan; 7 Fukuoka College of Health Sciences, Fukuoka, Japan

**Keywords:** Chronic liver diseases, Hepatic osteodystrophy, Reactive oxidative species, Stanniocalcin 1, Interleukin 17, Neutrophils

## Abstract

**Objective:**

Chronic liver diseases often involve metabolic damage to the skeletal system. The underlying mechanism of bone loss in chronic liver diseases remains unclear, and appropriate therapeutic options, except for orthotopic liver transplantation, have proved insufficient for these patients. This study aimed to investigate the efficacy and mechanism of transplantation of immature hepatocyte-like cells converted from stem cells from human exfoliated deciduous teeth (SHED-Heps) in bone loss of chronic liver fibrosis.

**Methods:**

Mice that were chronically treated with CCl_4_ received SHED-Heps, and trabecular bone density, reactive oxygen species (ROS), and osteoclast activity were subsequently analyzed *in vivo* and *in vitro*. The effects of *stanniocalcin 1* (*STC1*) knockdown in SHED-Heps were also evaluated in chronically CCl_4_ treated mice.

**Results:**

SHED-Hep transplantation (SHED-HepTx) improved trabecular bone loss and liver fibrosis in chronic CCl_4_-treated mice. SHED-HepTx reduced hepatic ROS production and *interleukin* 17 (*Il-17*) expression under chronic CCl_4_ damage. SHED-HepTx reduced the expression of both *Il-17* and *tumor necrosis factor receptor superfamily 11A* (*Tnfrsf11a*) and ameliorated the imbalance of osteoclast and osteoblast activities in the bone marrow of CCl_4_-treated mice. Functional knockdown of *STC1* in SHED-Heps attenuated the benefit of SHED-HepTx including anti-bone loss effect by suppressing osteoclast differentiation through TNFSF11–TNFRSF11A signaling and enhancing osteoblast differentiation in the bone marrow, as well as anti-fibrotic and anti-ROS effects in the CCl_4_-injured livers.

**Conclusions:**

These findings suggest that targeting hepatic ROS provides a novel approach to treat bone loss resulting from chronic liver diseases.

## Highlights

• Chronic CCl_4_ exposure enhances hepatic ROS levels and osteoclast activity.
• SHED-HepTx reduces hepatic ROS production caused by chronic CCl_4_ damage.
• SHED-HepTx reduced osteoclast activity caused by chronic CCl_4_ damage.
• Functional knockdown of *STC1* attenuates the benefit of SHED-HepTx.
• Targeting hepatic ROS is a novel approach for bone loss in chronic liver diseases.

## Graphical abstract

**Figure:**
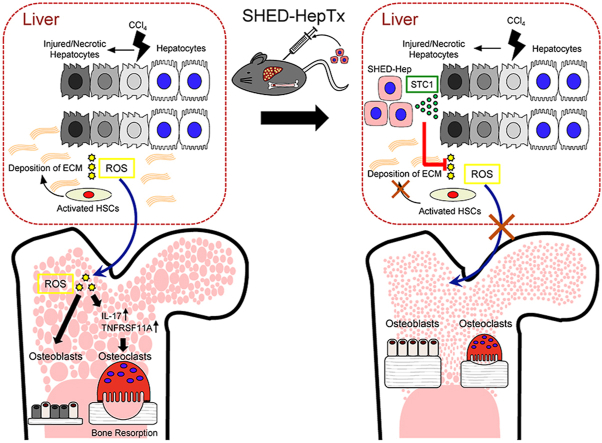

## Abbreviations

ACTA2: actin alpha 2, smooth muscle
ALT: alanine aminotransferase
AST: aspartate aminotransferase
BMCs: bone marrow cells
BMD: bone mineral density
Coll1a1: collagen type 1 alpha 1
CTX-I: C-terminal telopeptide of type I collagen
Ctsk: cathepsin K
ELISA: enzyme-linked immunosorbent assay
GSH-Px: glutathione peroxidase
HepPar1: human hepatocyte paraffin 1 antigen
HLA-ABC: human leukocyte antigens A, B, and C
HSCs: hepatic stellate cells
HYP: hydroxyproline
IL-17: interleukin 17
MDA: malondialdehyde
mHeps: mouse hepatocytes
microCT: microcomputed tomography
MNCs: multinuclear cells
MSCs: mesenchymal stem cells
NOX4: nicotinamide adenine dinucleotide phosphate oxidase 4
NFATc1: nuclear factor of activated T-cell
PBS: phosphate buffered saline
Pparg: peroxisome proliferator-activated receptor gamma
ROS: reactive oxygen species
RT-qPCR: quantitative reverse transcription polymerase chain reaction
SAA: serum amyloid A
SEM: standard error of the mean
SEMA3A: semaphorin 3A
SHED: stem cells from human exfoliated deciduous teeth
SHED-Heps: hepatocyte-like cells converted from stem cells from human exfoliated deciduous teeth
SHED-HepTx: SHED-Hep transplantation
siCONT: scrambled control siRNA
siRNA: small interfering RNA
siSTC1: siRNA specific for *STC1*
STC1: stanniocalcin 1
Th17 cells: T helper 17 cells
TNF: tumor necrosis factor
TNFSF11: TNF superfamily member 11
TNFRSF11A: TNF receptor superfamily member 11a
TRAP: tartrate resistant acid phosphatase

## 1 Introduction

The liver is a central organ possessing complex metabolic and xenobiotic functions in the digestive system, and it also participates in the endocrine system. Liver metabolism is highly involved in bone metabolism under physiological conditions through the function of somatotropic axis hormones such as growth hormone, insulin-like growth factor-I, and insulin-like growth factor binding protein 3 and of calciotropic hormones, including parathyroid hormone and vitamin D. Chronic liver diseases can potentially cause abnormal metabolism in the skeletal system [1]. The abnormal bone metabolism is associated with reduced bone mineral density (BMD) and decreased trabecular bone structures and induces bone loss likely due to osteoporosis or osteopenia [2]. Severe osteoporosis is frequently suffered in patients with chronic liver disease, especially in end-stages and in chronic cholestasis, non-alcoholic fatty liver disease, haemochromatosis, and alcoholism [1,2]. However, the pathogenesis and mechanisms underlying bone reduction in chronic liver disease remain unclear. Thus, it is necessary to elucidate the critical factors to develop an alternative option for bone loss in chronic liver disease.

Reactive oxygen species (ROS) are known to trigger the progression of chronic liver fibrosis [3]. ROS are released from injured hepatocytes and transform quiescent hepatic stellate cells (HSCs) into their active form. ROS-activated HSCs release several inflammatory cytokines that recruit immune cells into liver tissue. Among these inflammatory cytokines, interleukin 17 (IL-17) promotes HSCs to produce extracellular matrix in the context of liver fibrosis [4]. Thus, hepatic ROS function to trigger complex interactions between activated HSCs and recruited immune cells to exacerbate fibrosis and inflammation within the liver. However, the cellular mediators and molecular mechanisms of hepatic ROS-mediated bone loss in chronic liver fibrosis have not been elucidated.

Abnormal mineral turnover occurs due to the accelerated osteoclast function that underlies bone loss in patients with chronic liver disease [4,5]. Osteoclasts are responsible for bone mineral resorption and differentiated from bone marrow cells (BMCs) via tumor necrosis factor (TNF) receptor superfamily member 11a (TNFRSF11A) during osteoimmune communication [6]. IL-17 is known to induce osteoclasts via osteoblast; IL-17-related inflammatory conditions stimulate osteoblasts to express TNF superfamily member 11 (TNFSF11) and enhance osteoclast differentiation via TNFSF11–TNFRSF11A signaling [7]. Several chronic inflammatory diseases cause secondary bone reduction through IL-17-mediated osteoclasts [8,9], indicating a preventive potency of anti-IL-17 therapy in bone loss. However, it is not fully understood if hepatic ROS can trigger bone loss by IL-17-mediated osteoclasts in chronic liver fibrosis and whether targeting hepatic ROS is effective to bone loss, as well as hepatic dysfunction, in chronic liver fibrosis.

Human deciduous pulp stem cells were first identified tissue specific mesenchymal stem cells (MSCs) with clonogenicity properties with self-renewal and multipotency within the dental pulp tissues of exfoliated deciduous teeth and are referred to as stem cells from human exfoliated deciduous teeth (SHED) [10]. Current studies evaluated identified patient-derived dental pulp-tissue stem cells from disease-specific and disease-non-specific tissues [[11], [12], [13]] and clarify that the pharmacologically rejuvenation can improve dysfunction of patient-derived dental pulp-tissue stem cells [11,14,15]. Manufacturing of clinical grade SHED is established under a quality control for use in regenerative medicine [16,17]. Thus, SHED-based therapy is realistically considered to be a novel option for regenerative medicine [18]. Recently, it was reported that SHED that were converted into immature hepatocyte-like cells with limited hepatic functions (referred to as SHED-Heps) were established under the stimulation of hepatogenic cytokines and SHED-Hep transplantation (SHED-HepTx) showed anti-hepatic dysfunction and anti-fibrotic effects in animal models of Wilson’s disease, hemophilia A, and chronic liver fibrosis [[19], [20], [21]]. However, it remains unclear if SHED-Heps are a feasible option for bone loss in chronic liver fibrosis. The present study was designed to investigate the anti-bone loss therapy, as well as anti-hepatic dysfunction and anti-fibrotic therapies, of SHED-HepTx in chronic liver fibrosis. Furthermore, we examined whether targeting anti-hepatic ROS effect of SHED-HepTx is effective to bone loss, as well as hepatic dysfunction, in chronic liver fibrosis.

## 2 Materials and methods

### 2.1 Ethics statement, human subjects, and animals

Human deciduous teeth were collected from discarded clinical samples from healthy pediatric donors (5–7 years old, n = 3) with written informed consent from the guardian of each child donor at the Department of Pediatric Dentistry, Kyushu University Hospital. Procedures for handling human samples were approved by the Kyushu University Institutional Review Board for Human Genome/Gene Research (Protocol Numbers: 738-01, 02, 03, and 04). All animal experiments in this study were approved by the Institutional Animal Care and Use Committee of Kyushu University (protocol numbers: A20-041-0 and A21-044-1). All methods were performed in accordance with relevant guidelines and regulations.

### 2.2 Animals

C57BL/6J mice (female, 6 weeks old) and pregnant mice were obtained from the Jackson Laboratories Japan (Yokohama, Japan). The animals were housed individually and freely provided with sterile water and standard chow under controlled environmental conditions with a 12 h light/12 h dark cycle.

### 2.3 Culture of SHED and SHED-Heps

SHED were isolated by a colony-forming unit fibroblast method, cultured, and characterized as previously described [22]. SHED-Heps were induced under hepatogenic conditions and characterized as previously described [16,19]. SHED-Heps were pretreated for 3 days with small interfering RNA (siRNA) specific for *stanniocalcin 1* (*STC1*) or with scrambled control (referred to as siSTC1 and siCONT, respectively; Santa Cruz Biotechnology, Santa Cruz, CA, USA). The culture details are described in the Supplementary Methods.

### 2.4 SHED-HepTx into chronically CCl_4_-treated liver fibrosis model mice

Freshly prepared CCl_4_ (1 mg/kg diluted 1:4 in olive oil; Nacalai Tesque, Kyoto, Japan), was intraperitoneally injected into mice twice a week. SHED-Heps (1 × 10^6^ in 100 μL phosphate buffered saline [PBS]) or PBS control (100 μL) were transplanted into 4-week CCl_4_-treated mice via the spleen, and additional CCl_4_ was administered for four weeks (referred to as SHED-HepTx mice and CCl_4_ mice, respectively. Age-matched mice received olive oil alone (Nacalai Tesque), referred to as control mice. All animals did not receive any immunosuppression and conditioning throughout this study. Mouse livers, long bones, and serum were harvested eight weeks after CCl_4_ treatment.

### 2.5 *In vivo* fibroinflammatory and ROS production assays in CCl_4_ treated mice

Serum levels of aspartate aminotransferase (AST), alanine aminotransferase (ALT), total bilirubin, and hepatic hydroxyproline (HYP) were measured by colorimetric analyses. The hepatic distribution of collagen was analyzed by Sirius Red staining. The hepatic localization of actin alpha 2 and smooth muscle (ACTA2) was analyzed by immunohistochemical analysis. The hepatic expression of *Acta2*, *collagen type 1 alpha 1* (*Coll1a1*), *Il-17*, *peroxisome proliferator-activated receptor gamma* (*Pparg*), and *nicotinamide adenine dinucleotide phosphate oxidase 4* (*Nox4*) was analyzed by quantitative reverse transcription polymerase chain reaction (RT-qPCR). Malondialdehyde (MDA) levels and glutathione peroxidase (GSH-Px) activity were measured in mouse livers by colorimetric analysis. The expression of *serum amyloid A1* (*Saa1*) in mouse livers were analyzed by RT-qPCR. Serum levels of granulocyte stimulating factor (G-CSF), IL-17, SAA1, and transforming growth factor beta (TGFB) were analyzed using ELISA.

### 2.6 *In vivo* monitoring of donor cells in CCl_4_-treated mice

SHED-Heps were labeled with XenoLight DiR NIR fluorescent dye (DiR; 10 μg/mL; Perkin Elmer, Waltham, MA; 1 × 10^6^ in 100 μL PBS) or PBS (100 μL) and then intrasplenically infused into 4-week-CCl_4_ treated mice. Ventral images of the mice were obtained 24 h after infusion with IVIS Lumina III (Perkin Elmer) using living image software (Perkin Elmer).

### 2.7 Immunological localization of donor cells in CCl_4_-treated mice

The hepatic distribution of human leukocyte antigens A, B, and C (HLA-ABC) and of human hepatocyte paraffin 1 antigen (HepPar1) was analyzed by immunohistochemical analysis. The co-distribution of HepPar1 and ACTA2 in mouse livers was analyzed using double immunofluorescence analysis.

### 2.8 Bone mineral assays in CCl_4_-treated mice

Trabecular bones of mouse tibiae were analyzed by micro-computed tomography (microCT) assays performed on a SkyScan 1076 scanner (Bruker, Billerica, MA, USA) using CT-Analyzer and CT-Volume software (Bruker) [23]. Serum levels of C-terminal telopeptide of type I collagen (CTX-I) and tartrate-resistant acid phosphatase 5 b (TRAP-5b) were analyzed by an enzyme-linked immunosorbent assay (ELISA).

### 2.9 *In vitro* osteoclast inductive assay

Mouse BMCs were isolated from femurs and tibiae of mice and co-cultured with newborn calvarial osteoblasts and the osteoclast formation was determined as reported previously [24,25], as described in the Supplementary Methods. Some co-cultures were incubated with CCl_4_ (2 μg/mL, Nacalai Tesque) or recombinant mouse IL-17 (10 nM, PeproTech, Cranbury, NJ, USA) and/or anti-mouse TNFSF11 goat IgG (50 ng/mL, R&D Systems, Minneapolis, MN, USA) or goat IgG (50 ng/mL, R&D Systems). Mouse BMCs were treated for 4 days in the absence and presence of H_2_O_2_ (0.1 mM diluted in PBS, Nacalai Tasque). The expression of *Il-17* and *Tnfrsf11a* in mouse BMCs was analyzed by RT-qPCR.

### 2.10 *In vitro* osteoblast inductive assay

Mouse bone marrow stromal cells (BMSCs) were cultured under an osteogenic induction condition, and determined as previously [25], as described in the Supplementary Methods. Some osteogenic cultures were incubated with CCl_4_ (2 μg/mL, Nacalai Tesque) or H_2_O_2_ (0.1 mM diluted in PBS, Nacalai Tasque).

### 2.11 *In vitro* ROS production and anti-oxidative stress assays

Primary mouse hepatocytes (mHeps, 3 × 10^5^ per well) were incubated with or without CCl_4_ (2 μg/mL; Nacalai Tesque) for 4 h and co-cultured with or without SHED-Heps (0.1, 0.2, 1.0, and 2.0 × 10^5^ per well) using 0.4 μm cell culture inserts (Thermo Fisher Scientific, Waltham, MA, USA) for 6 h in 10% fetal bovine serum (Equitech-Bio, Kerrville, TX, USA), 5% non-essential amino acids (Nacalai Tesque), and premixed antibiotics containing 100 U/mL penicillin and 100 μg/mL streptomycin (Nacalai Tesque) in Dulbecco’s modified Eagle’s medium (Nacalai Tesque). The ROS content in the conditioned medium and the cell viability of mHeps were both analyzed by colorimetry.

### 2.12 Statistical analysis

Each test was performed in triplicate, and the results were expressed as the mean ± standard error of the mean. Comparisons between two groups were performed using independent two-tailed Student’s t-test. Multiple group comparisons were performed using one-way repeated measures analysis of variance followed by the Tukey’s post hoc test. Statistical significance was set at P *˂* 0.05. All statistical analyses were performed using PRISM 6 software (GraphPad Software, La Jolla, CA, USA).

## 3 Results

### 3.1 Donor SHED-Heps engraft into the periportal area of CCl_4_-injured mouse livers

Isolated SHED exhibited characteristics of MSCs, including attached colony formation, immunophenotypes, and mesenchymal multipotency (Supplementary Figure 1). SHED-Heps exhibited immature hepatocyte-like characteristics and limited-hepatic function compared to primary human hepatocytes by hepatocyte-specific gene expression and hepatic function analyses (Supplementary Figure 2).

**Figure 1:**
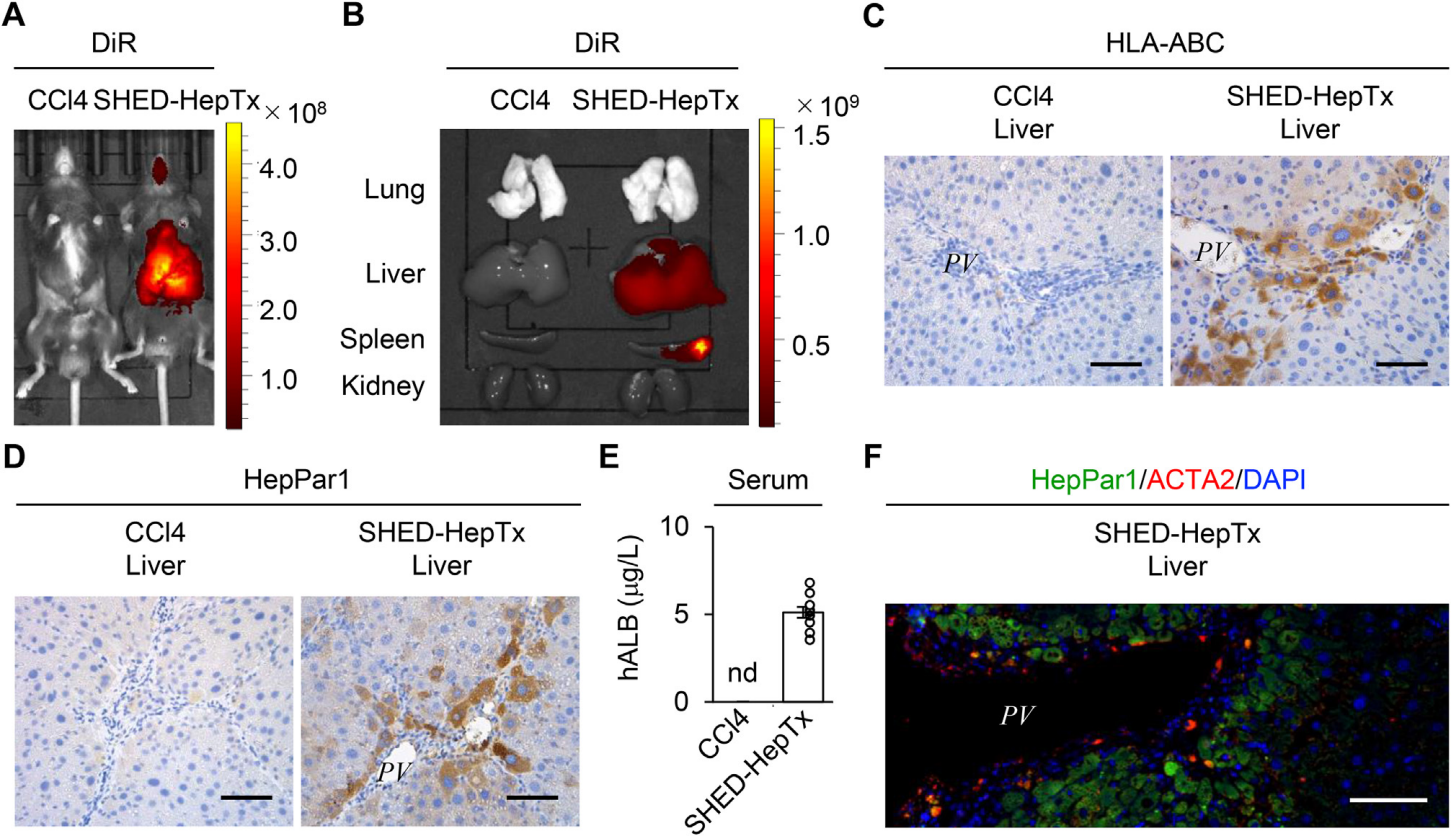
**Donor SHED-Heps engraft into the periportal area of CCl**_**4**_**-injured mouse livers.** (A, B) DiR-labeled SHED-Heps were intrasplenically transplanted into CCl_4_-treated mice. Representative images of DiR-labeled donor SHED-Heps were detected for the whole bodies (A) and for lungs, livers, spleens, and kidneys (B) of CCl_4_-treated mice five days after infusion by *in vivo* image analysis. (C–F) Mice were harvested four weeks after transplantation of SHED-Heps (SHED-HepTx). Representative liver images of human leukocyte antigen A, B, and C (HLA-ABC) (C) and human hepatocyte paraffin 1 (HepPar1) (D) were by immunohistochemical analysis. The graph presents the serum levels of human albumin (hALB) by ELISA (E). A representative liver image of HepPar1 and ACTA2 was detected by double immunofluorescent analysis (F). A–F: CCl4, CCl_4_-treated group; SHED-HepTx, SHED-HepTx group. C, D, F: Nuclei were stained with hematoxylin (C, D) and 4′,6-diamidino-2-phenylindole (DAPI; F). *PV*: portal vein. Scale bars, 50 μm (C, D, F). E: n = 10 for all groups. ∗*P ˂*  0.05, ∗∗*P ˂*  0.01, ∗∗∗*P ˂*  0.005. nd, no detection; ns, no significance. The graph bars represent the means ± standard error of the mean (SEM).

*In vivo* imaging demonstrated that DiR-fluorescence activity was detected the donor cell *in situ* in the liver region of DiR-labeled SHED-HepTx mice but not in that of the non-infused mice at 5 days after infusion (Figure 1A). Using *ex vivo* imaging, DiR-fluorescence activity was detected in the livers and spleens but not in the lungs and kidneys of the DiR-labeled SHED-HepTx mice (Figure 1B). No fluorescent activity was detected in the lung, livers, spleens, and kidneys of non-infused mice (Figure 1B). Immunohistochemical analysis revealed that HLA-ABC-positive and HepPar1-positive cells were engrafted into the periportal region of the livers of SHED-HepTx mice (SHED-HepTx livers) but not in the livers of control and CCl_4_ mice (control and CCl_4_ livers, respectively) (Figure 1C, D). ELISA detected serum human albumin in the SHED-HepTx mice but not in the control and CCl_4_ mice (Figure 1E). Double immunofluorescence analysis demonstrated that donor HepPar1-positive cells were localized close to recipient ACTA2-positive cells in the periportal region of SHED-HepTx livers (Figure 1F). Immunohistochemical control tests using mouse IgG1 and IgG2a revealed no immune reactions (Supplementary Figure 3).

### 3.2 SHED-HepTx improves hepatic ROS-induced fibro-inflammation in CCl_4_-injured mice

**Figure 2:**
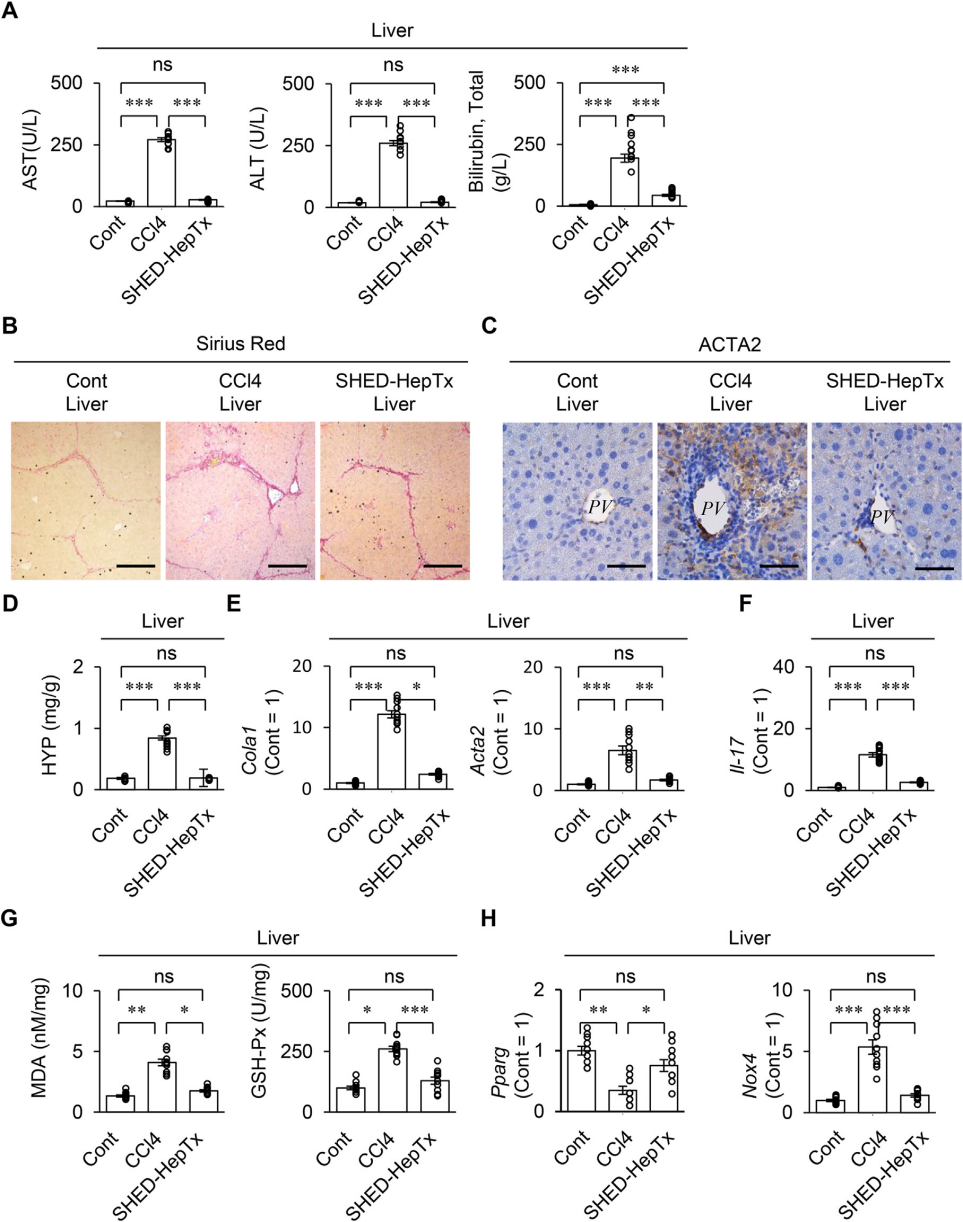
**SHED-HepTx improves the hepatic fibro-inflammation and suppresses the production of reactive oxygen species (ROS) in CCl**_**4**_**-treated mice.** CCl_4_-treated mice were harvested four weeks after SHED-HepTx. (A) The graphs present the serum levels of aspartate aminotransferase (AST), alanine aminotransferase (ALT), and total bilirubin by colorimetric analysis. (B) Representative liver images were analyzed by Sirius red staining. (C) Representative liver images of actin alpha 2 and smooth muscle (ACTA2) were acquired by immunohistochemical analysis. Nuclei were stained with hematoxylin. *PV*, portal vein. (D) The graph indicates the hepatic hydroxyproline (HYP) content by colorimetric analysis. (E) Graphs presenting the hepatic expression of *collagen type I alpha chain* (*Col1a*) and *Acta2* by RT-qPCR. (F) The graphs indicate the hepatic *interleukin 17 (Il-17)* expression (H) by RT-qPCR (G) The graphs present the hepatic levels of malondialdehyde (MDA) and glutathione peroxidase (GSH-Px) by colorimetric analysis. (H) The graphs indicate the hepatic expression of *peroxisome proliferator-activated receptor gamma (Pparg) and nicotinamide adenine dinucleotide phosphate oxidase 4 (Nox4)* by RT-qPCR. A–H: Cont, olive oil-treated group; CCl4, CCl_4_-treated group; SHED-HepTx, SHED-HepTx group A, D–H: n = 10 for all groups. ∗*P ˂*  0.05, ∗∗*P ˂*  0.01, ∗∗∗*P ˂*  0.005. ns, no significance. The graph bars represent the mean ± SEM. B, C: Scale bars: 200 μm (B) and 50 μm (C). E, F, H: The results are presented as a ratio of the expression to that of the control group (Cont = 1).

Eight weeks after CCl_4_ treatment, the CCl_4_ mice exhibited increased liver fibrosis compared to the control mice (Figure 2). The SHED-HepTx mice exhibited the reduced serum levels of AST, ALT, and total bilirubin compared to the CCl_4_ mice by colorimetric analyses (Figure 2A). By Sirius Red staining and immunohistochemical analysis, the SHED-HepTx livers reduced the deposition of fibrous tissues and expression of ACTA2 compared to the CCl_4_ livers (Figure 2B,C). By colorimetric analysis and RT-qPCR, the SHED-HepTx livers decreased the HYP content and expression of *Acta2* and *Col1a1* compared to the CCl_4_ livers (Figure 2D, E).

Hepatic ROS production is consistently manifested by measuring MDA and GSH-Px in liver [26]. *Pparg* and *Nox4* are unambiguous markers of activated HSCs under ROS stimulation [27,28]. IL-17 is a critical mediator to produce extracellular matrix by HSCs [4]. RT-qPCR revealed that the CCl_4_ livers showed higher *Il-17* expression than the control livers, while the SHED-HepTx livers expressed lower *Il-17* level than the CCl_4_ livers (Figure 2F). The enhanced ROS production and increased HSC activation was determined in the CCl_4_ livers compared to the control livers, as indicated by the increased levels of MDA and GSH-Px by colorimetric analysis and the reduced *Pparg* and increased *Nox4* levels by RT-qPCR, meanwhile, the SHED-HepTx livers suppressed the ROS production and HSC activation (Figure 2G, H).

### 3.3 SHED-HepTx improves trabecular bone density and suppresses osteoclast differentiation via IL-17 in the bone marrow of CCl_4_-injured mice

**Figure 3:**
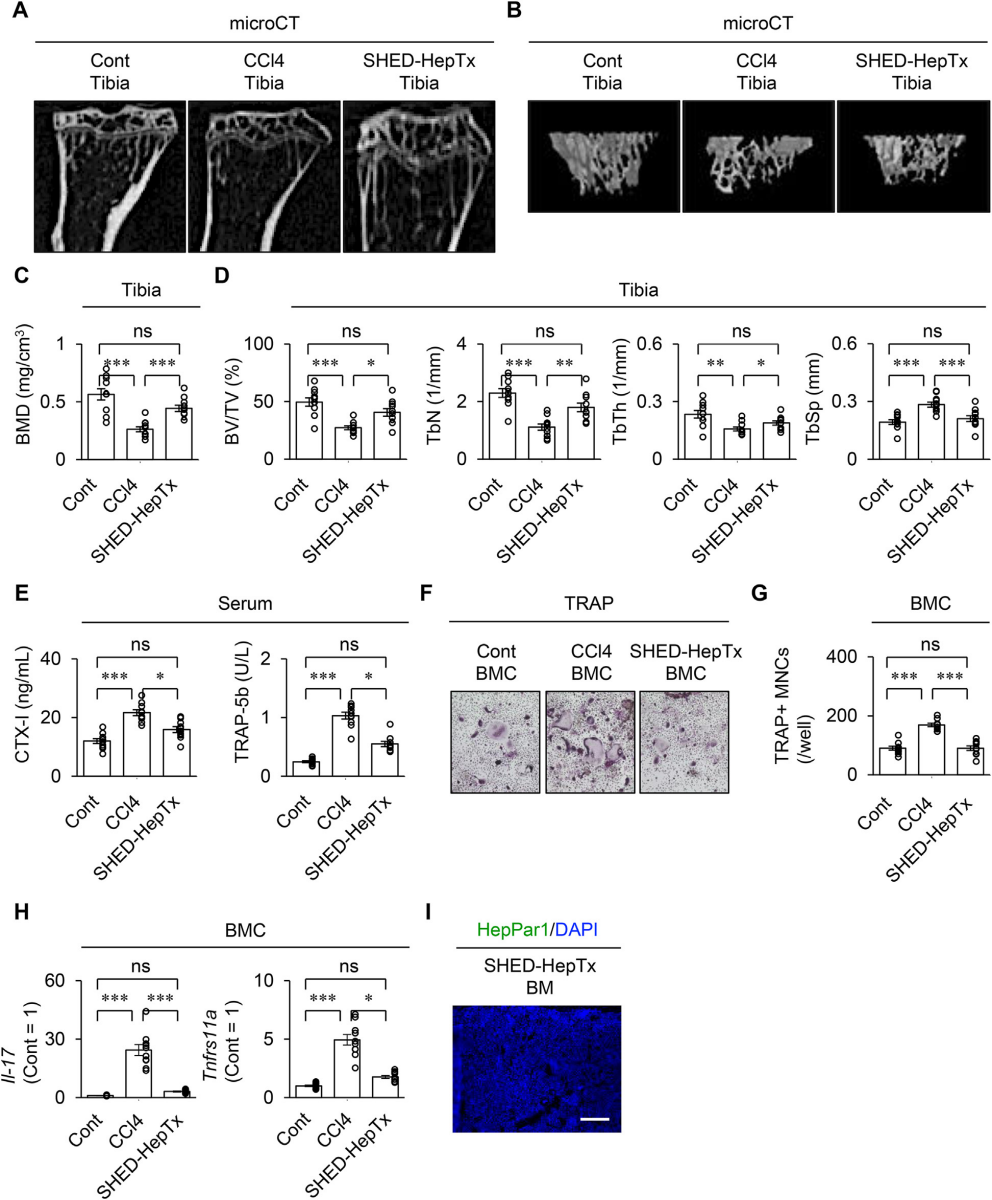
**SHED-HepTx recovers bone density and suppresses osteoclast differentiation in CCl**_**4**_**-treated mice.** Mouse long bones were harvested four weeks after SHED-HepTx. (A–C) Representative images presenting two- (2D) (A) and three-dimensional (3D) (B) proximal tibiae by micro-computed tomographic (microCT) analysis. The graphs present bone mineral density (BMD) (C) and bone parameters. BV/TV, bone volume/total volume; TbN, trabecular number; TbTh, trabecular thickness; TbSp, trabecular spacing (D). (E) The graphs provide the serum levels of C-terminal fragments of type I collagen (CTX-I) and tartrate-resistant acid phosphatase 5 b (TRAP-5b) by ELISA. (F, G) Mouse bone marrow cells (BMCs) were co-cultured with calvarial osteoblasts. Representative images of osteoclast differentiation were analyzed by TRAP staining (F). The graph indicates the number of TRAP-positive (TRAP^+^) multinuclear cells (MNCs; G). (H) The graphs indicate the expression of *Il-17* and *tumor necrosis factor receptor superfamily 11a* (*Tnfrsf11a*) in BMCs by RT-qPCR. The results are presented as a ratio to the expression in the control group (Cont = 1). (I) A representative bone marrow image of HepPar1 was analyzed by immunofluorescent analysis. Nuclei were stained with DAPI. Scale bars, 300 μm. A–I: Cont, olive oil-treated group; CCl4, CCl_4_-treated group; SHED-HepTx, SHED-HepTx group. C–E, G–I: n = 10 for all groups. The graph bars represent the mean ± SEM. ∗*P*  *˂*  0.05, ∗∗*P*  *˂*  0.01, ∗∗∗*P*  *˂*  0.005. ns, no significance.

MicroCT analysis revealed that the femurs of CCl_4_ mice exhibited decreased trabecular bone structure, reduced trabecular BMD and abnormal trabecular bone parameters, including bone volume/trabecular volume, trabecular thickness, trabecular number, and trabecular separation, compared to that of the control mice (Figure 3A–D). ELISA demonstrated that the CCl_4_ mice expressed the increased serum levels of CTX-I and TRAP-5b compared to the control mice (Figure 3E). BMCs were isolated from control, CCl_4_-treated, and SHED-Hep-Tx mice (Cont-BMCs, CCl_4_-BMCs, and SHED-Hep-TX-BMCs, respectively) and cocultured with calvarial osteoblasts. *In vitro* osteoclastogenic assay revealed that the CCl_4_-BMCs increased the number of TRAP-positive multinuclear cells (MNCs) and levels of *Tnfrsf11a*, *nuclear factor of activated T-cell* (*Nfatc1*), and *cathepsin K* (*Ctsk*), compared to the Cont-BMCs by TRAP staining and RT-qPCR (Figure 3F, G and Supplementary Figure 4). Conversely, the SHED-HepTx mice exhibited recovery of bone structure, BMD, and parameters of trabecular bone and improved serum levels of CTX-I and TRAP-5b compared to the CCl_4_ mice by microCT analysis and ELISA (Figure 3A–E). *In vitro* osteoclastogenic assay revealed that the SHED-HepTx-BMCs exhibited lower osteoclast differentiation than the CCl_4_-BMCs by TRAP staining and RT-qPCR (Figure 3F, G and Supplementary Figure 4). Moreover, the CCl_4_-BMCs increased the expression of *Il-17* and *Tnfrsf11a* compared to the Cont-BMCs, while the SHED-HepTx-BMCs possessed lower expression of *Il-17* and *Tnfrsf11a* than the CCl_4_-BMCs, by RT-qPCR (Figure 3H).

Mouse BMSCs were isolated from control, CCl_4_, and SHED-Hep-Tx mice (Cont-BMSCs, CCl_4_-BMSCs, and SHED-HepTx-BMSCs, respectively). *In vitro* osteogenic assay revealed that the CCl_4_-BMSCs reduced the osteogenic capacity compared to the Cont-BMSCs, as indicated by the decreased formation of mineralized nodules and suppressed expression of *runt-related transcription factor 2*, alkaline phosphatase, and *bone gamma-carboxyglutamate protein*, four and two weeks after osteogenic induction by Alizarin Red staining and RT-qPCR, respectively (Supplementary Figure 5). Meanwhile, the SHED-HepTx-BMSCs showed the improved osteogenic capacity compared to the CCl_4_-BMSCs (Supplementary Figure 5). Immunofluorescence analysis showed that HLA-ABC-positive cells were not detected in the bone marrow of SHED-HepTx mice four weeks after transplantation (Figure 3I), indicating that the bone marrow is not the direct target of donor SHED-Heps.

### 3.4 Bone cells is impaired by ROS, but not by CCl_4_, *in vitro*

Since CCl_4_ causes necrosis of hepatocytes [29], the toxicity of CCl_4_ to mouse BMCs and BMSCs were analyzed under the stimulation of CCl_4_. The CCl_4_ stimulation induced no cell death of both BMCs and BMSCs by colorimetric assay (Supplementary Figure 6) and showed no effects on the osteoclastogenic and osteogenic capacities of BMCs and BMSCs by *in vitro* osteoclastogenic and osteogenic assays (Supplementary Figures 7 and 8).

The effects of ROS on mouse BMCs and BMSCs were analyzed under the stimulation of H_2_O_2_. The H_2_O_2_ stimulation enhanced the expression of *Il-17* and *Tnfrsf11a* in BMCs by RT-qPCR (Supplementary Figure 9). The H_2_O_2_ stimulation suppressed the osteogenic functions of BMSCs by Alizarin Red staining and RT-qPCR (Supplementary Figure 10) and inhibited the expression of *semaphorin 3a* (*Sema3a*) by RT-qPCR (Supplementary Figure 10E).

### 3.5 siSTC1-SHED-HepTx attenuates the suppression of hepatic oxidative stress and anti-fibroinflammatory effects in CCl_4_-damaged mouse livers

**Figure 4:**
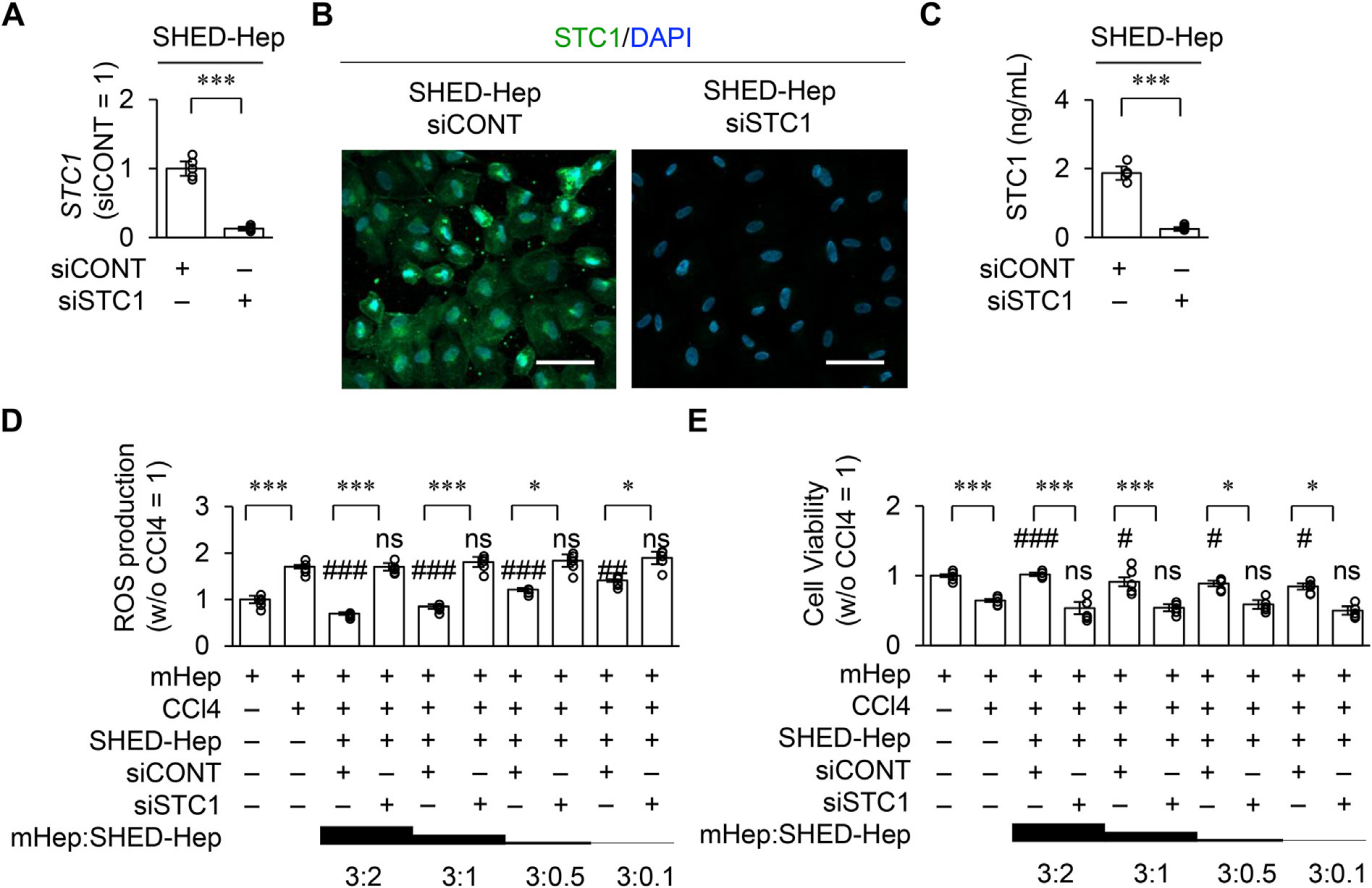
**Stanniocalcin 1 (STC1) knock-down attenuates the effects of SHED-Heps on CCl**_**4**_**-induced ROS production and cell survival of mouse hepatocytes (mHeps).** SHED-Heps were treated with siRNA specific for *STC1* (siSTC1) and scrambled control siRNA (siCONT), referred to as siSTC1-SHED-Heps and siCONT-SHED-Heps. (A) The graph presents the expression of *STC1* in SHED-Heps by RT-qPCR. The results are presented as a ratio of the expression in siCONT-SHED-Heps (siCONT = 1). (B) Representative images of STC1 in SHED-Heps were detected by immunofluorescence analysis. The nuclei were stained with DAPI. Scale bar, 30 μm. (C) The graph shows the levels of STC1 in the conditioned medium of SHED-Heps by ELISA. (D, E) mHeps were co-cultured with SHED-Heps under the stimulation of CCl_4_ (2 μg/mL). The graph presents the production of ROS (D) and cell viability (E) in mHeps by colorimetric analysis. The results are presented as a ratio of the expression in mHeps without CCl_4_ stimulation (w/o CCl4 = 1). A, C–E: n = 5 for all groups. ∗*P ˂*  0.05, ∗∗∗*P ˂*  0.005. #*P ˂*  0.05, ##*P ˂*  0.01, ###*P ˂*  0.005. (vs. CCl_4_-treated mHeps). ns, no significant difference (vs. CCl_4_-treated mHeps). The graph bars represent the mean ± SEM.

STC1 reduces ROS levels via mitochondrial uncoupling protein-2 in mammalian cells [30]. SHED-Heps were treated with siSTC1 and siCONT (siSTC-SHED-Heps and siCONT-Heps, respectively). The efficacy of siSTC1 treatment was confirmed by RT-qPCR, immunofluorescence analysis, and ELISA (Figure 4A–C). The CCl_4_ stimulation induced the ROS production and cell death of mHeps *in vitro* (Figure 4D, E). When mHeps were indirectly co-cultured with siCONT-SHED-Heps or siSTC-SHED-Heps under CCl_4_ stimulation, siCONT-SHED-Heps decreased the ROS production and cell death of mHeps but siSTC1-SHED-Heps diminished the effects of siCONT-SHED-Heps to mHeps (Figure 4D, E).

**Figure 5:**
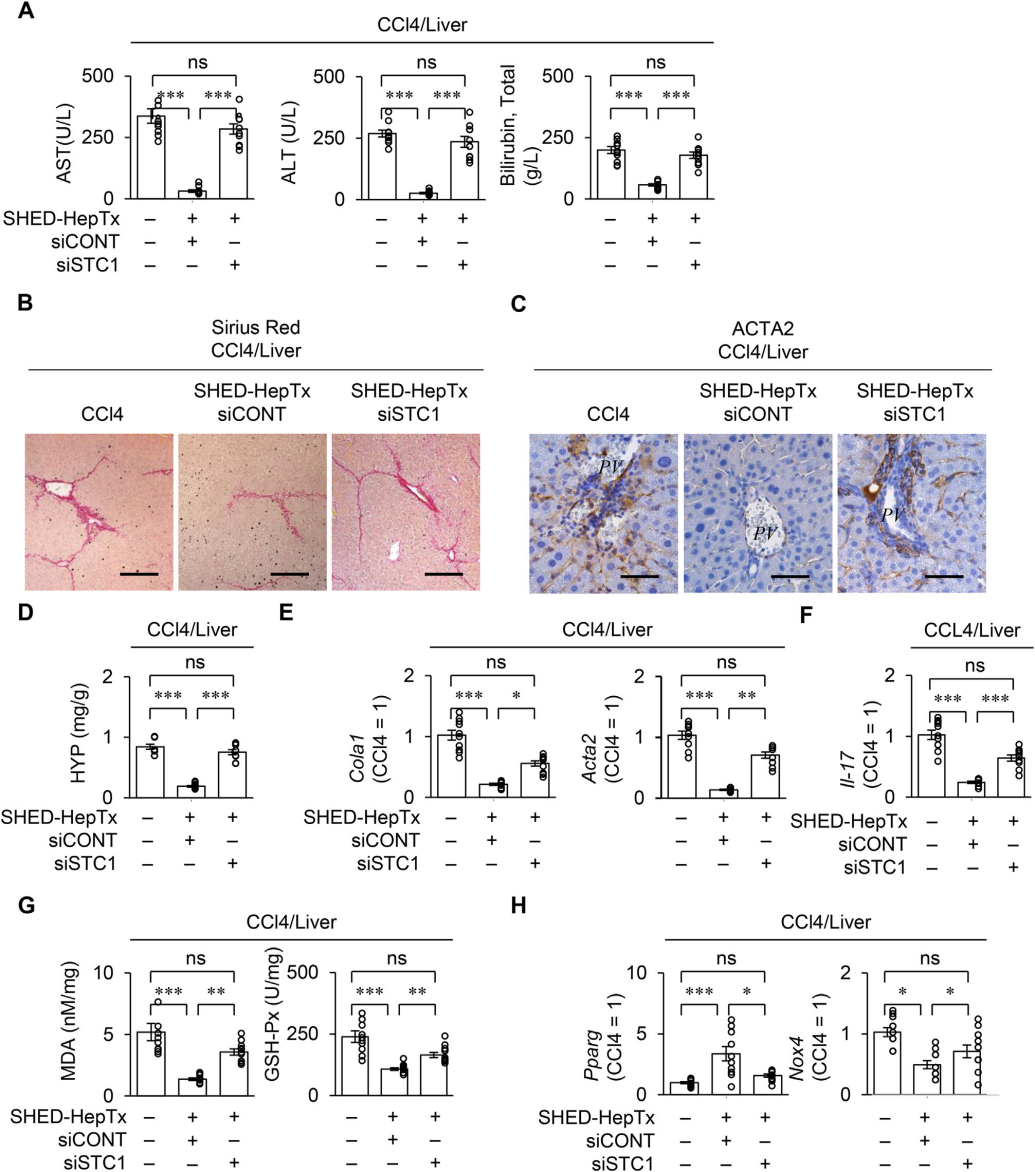
**STC1 knock-down attenuates the effects of SHED-HepTx on the anti-fibro-inflammation and ROS production in CCl**_**4**_**-treated mice.** CCl_4_-treated mice were harvested four weeks after SHED-HepTx. (A) The graphs present the serum levels of AST, ALT, and total bilirubin by colorimetric analysis. (B, C) Representative liver images were analyzed by Sirius Red staining (B). Representative liver images of ACTA2 were acquired by immunohistochemical analysis. Nuclei were stained with hematoxylin (C). Scale bars: 200 μm (B) and 50 μm (C). *PV*: portal vein. (D) The graph presents the hepatic HYP content by colorimetric analysis. (E) The graphs present the hepatic expression of *Col1a1* and *Acta2* by RT-qPCR. (F) The graphs present the hepatic expression of *Il-*17 by RT-qPCR. (G) The graphs present the hepatic levels of MDA and GSH-Px livers by colorimetric analysis. (H) The graphs present the hepatic expression of *Pparg* and *Nox4* by RT-qPCR. The results are presented as a ratio of the expression in the CCl_4_ group (CCl4 = 1). A–H: CCl4, CCl_4_-treated group; SHED-HepTx, SHED-HepTx group; siCONT, siCONT treatment; siSTC1, siSTC1 treatment. A, D–H: n = 10 in all groups. ∗*P ˂*  0.05, ∗∗*P ˂*  0.01, ∗∗∗*P ˂*  0.005. ns, no significance. The graph bars represent the mean ± SEM. E, F, H: The results are presented as a ratio of the expression in the CCl_4_-treated group (CCl4 = 1).

Next, siSTC1-SHED-Heps were transplanted into CCl_4_-treated mice (siSTC1-SHED-HepTx mice) and compared to siCONT-SHED-Hep transplanted CCl_4_-treated mice (siCONT-SHED-HepTx mice) to examine the benefit of STC1 to liver fibrosis. The siCONT-SHED-HepTx mice improved the hepatic anti-fibroinflammatory effects compared to the CCl_4_ mice (Figure 5). Meanwhile, the siSTC1-SHED-HepTx mice possessed the increased levels of serum AST, ALT, total bilirubin, hepatic fibrous tissue, and hepatic ACTA2 compared to the siCONT-SHED-HepTx mice by colorimetric assay, Sirius Red staining, and immunohistochemical analysis (Figure 5A–C). The livers of siSTC1-SHED-HepTx mice (siSTC1-SHED-HepTx livers) diminished the levels of HYP, *Col1a1*, and *Acta2* in the livers of siCONT-SHED-HepTx mice (siCONT-SHED-HepTx livers) by colorimetric analysis and RT-qPCR (Figure 5D, E). The siSTC1-SHED-HepTx livers exhibited the increased expression of *Il-17* compared to the siCONT-SHED-HepTx livers by RT-qPCR (Figure 5F). By colorimetric assays and RT-qPCR, the siSTC1-SHED-HepTx livers increased the ROS production and ROS-mediated HSC activation associated with the abnormal expression of MDA, GSH-Px, *Pparg*, and *Nox4* compared to the siCONT-SHED-HepTx livers (Figure 5G, H).

The CCl_4_ mice enhanced the levels of hepatic *Saa1* and serum SSA1, G-CSF, and IL-17 compared to the control mice by RT-qPCR and ELISA (Supplementary Figure 11). siCONT-SHED-HepTx improved the levels of hepatic *Saa1* and serum SAA1, G-CSF, and IL-17 in the CCl_4_ mice, while siSTC1-SHED-HepTx attenuated the benefit of siCONT-SHED-HepTx in the CCl_4_ mice (Supplementary Figure 11).

### 3.6 siSTC1-SHED-HepTx attenuates trabecular bone recovery and induces osteoclast differentiation via IL-17 in the bone marrow of CCl_4_-injured mice

**Figure 6:**
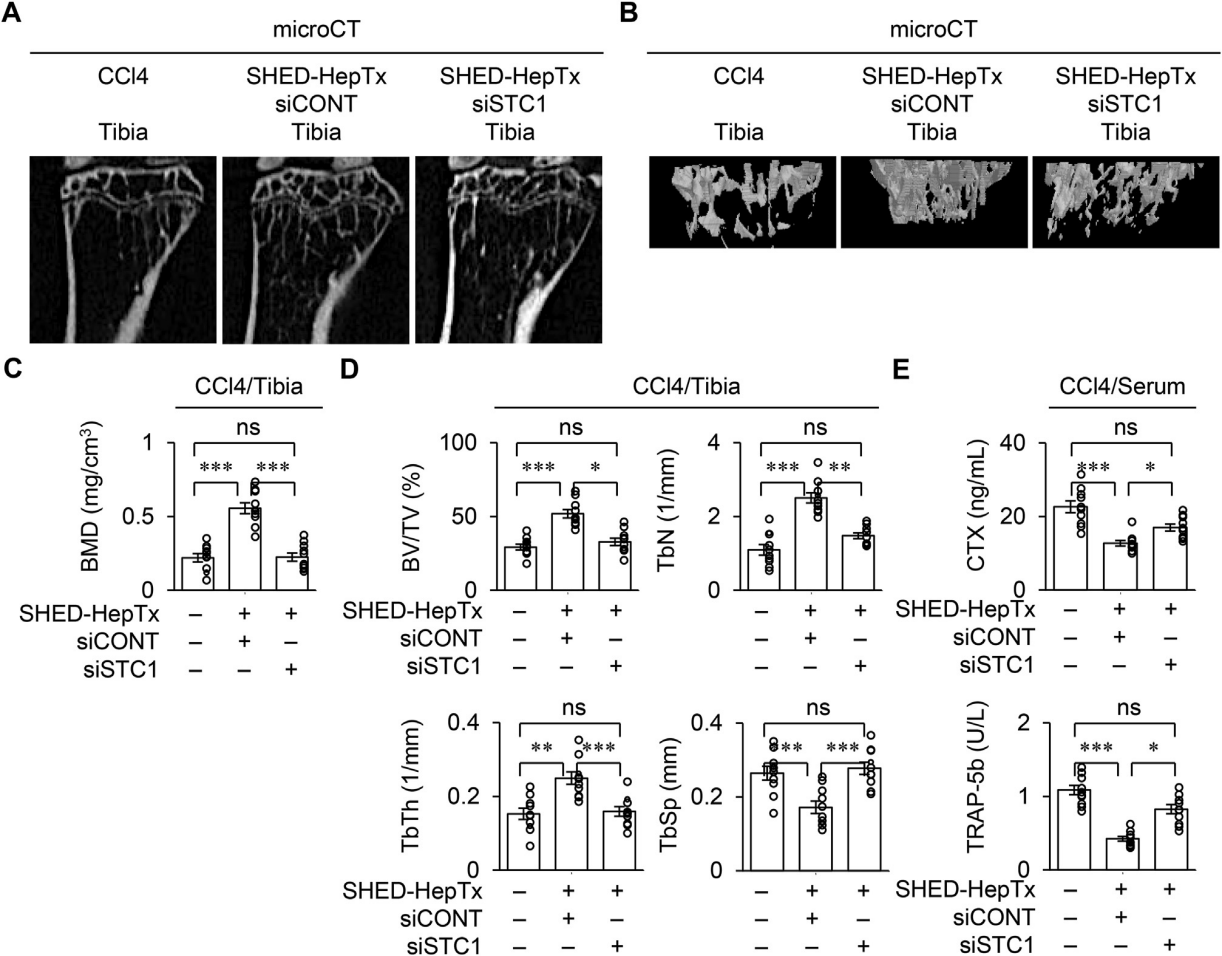
**STC1 knock-down attenuates the bone recovery in CCl**_**4**_**-treated mice with SHED-HepTx.** CCl_4_-treated mice were harvested four weeks after SHED-HepTx. (A–D) Representative 2D (A) and 3D (B) images of proximal tibiae by microCT analysis. The graphs present BMD (C) and bone parameters (D). (E) The graphs present the serum levels of CTX-I and TRAP-5b by ELISA. A–E: CCl4, CCl_4_-treated group; SHED-HepTx, SHED-HepTx group; siCONT, siCONT treatment; siSTC1, siSTC1 treatment. C–E: n = 10 for all groups. ∗*P ˂* 0.05, ∗∗*P ˂* 0.01, ∗∗∗*P ˂* 0.005. ns, no significance. The graph bars represent the mean ± SEM.

**Figure 7:**
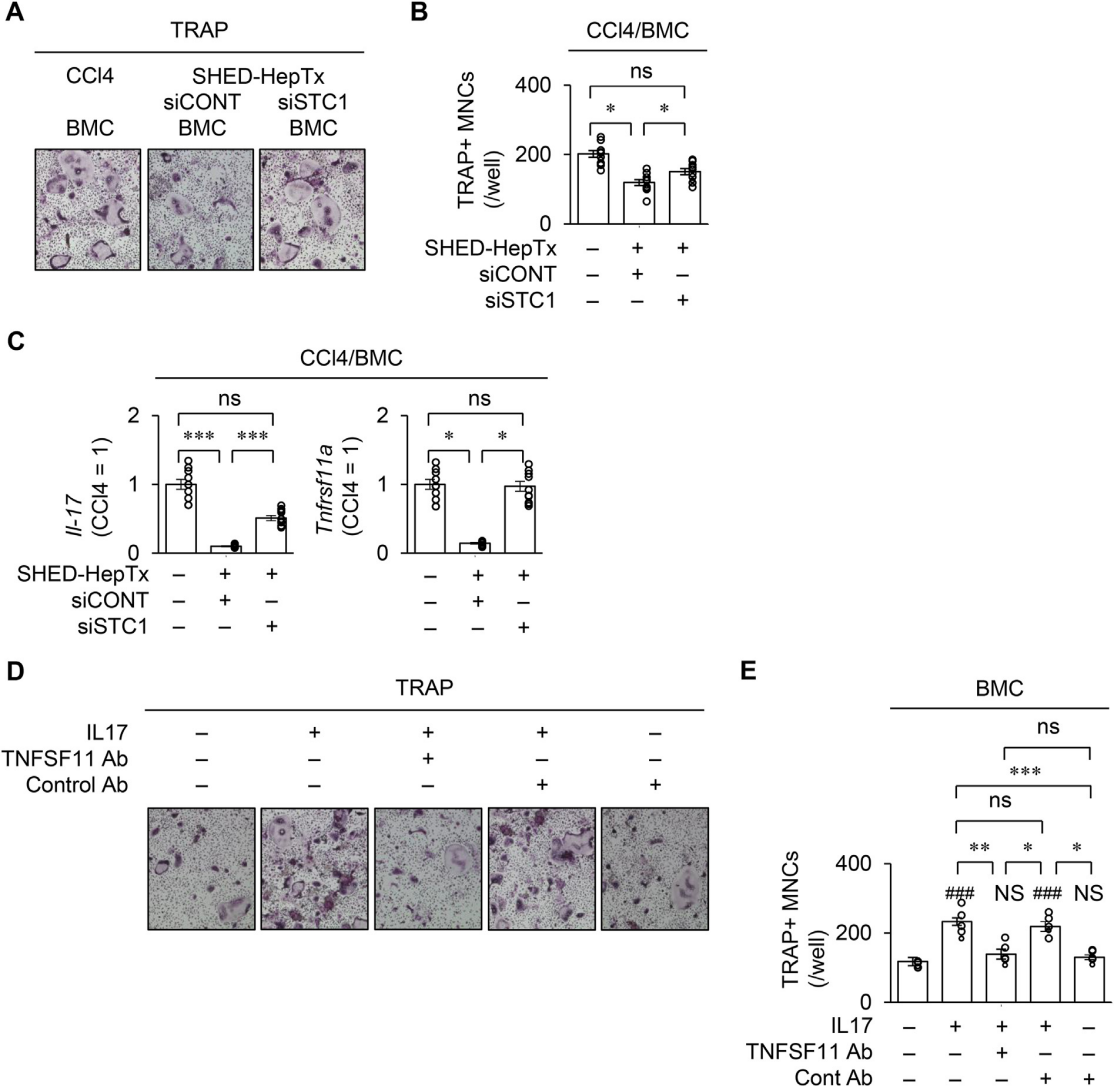
**STC1 knock-down attenuates the suppression of osteoclast differentiation via IL-17 enhanced tumor necrosis factor superfamily 11 (TNFSF11) in CCl**_**4**_**-treated mice with SHED-HepTx.**BMCs were co-cultured with calvarial osteoblasts. (A–C) CCl_4_-treated mice were harvested four weeks after SHED-HepTx. Representative images of osteoclast differentiation were analyzed by TRAP staining (A). The graph presents the number of TRAP^+^ MNCs. (B). The graphs indicate the expression of *Il-17* and *Tnfrsf11a* in BMCs according to RT-qPCR. The results are presented as a ratio to the expression in the CCl_4_ group (CCl4 = 1) (C). (D, E) The co-cultures were treated with or without recombinant mouse IL-17 (10 nM) and/or anti-mouse TNFSF11 goat IgG (TNFSF11 Ab; 50 ng/mL) or its control IgG antibody (Cont Ab). Representative images of osteoclast differentiation were analyzed by TRAP staining (D). The graph presents the number of TRAP^+^ MNCs. n = 5 for all groups. ###*P ˂* 0.005 vs. control group without IL-17, TNFSF11 Ab, and Cont Ab treatment NS, no significance vs. control group. (E). A–C: CCl4, CCl_4_-treated group; SHED-HepTx, SHED-HepTx group; siCONT, siCONT treatment; siSTC1, siSTC1 treatment. B, C, E: ∗*P ˂* 0.05, ∗∗*P ˂* 0.01, ∗∗∗*P ˂* 0.005. ns, no significance. The graph bars represent the mean ± SEM. B, C: n = 10 for all groups.

siCONT-SHED-HepTx exhibited the anti-bone loss effects in the CCl_4_ mice (Figure 6). siSTC1-SHED-HepTx attenuated the benefit of siCONT-SHED-HepTx by microCT analysis and ELISA (Figure 6A–E). *In vitro* osteoclastogenic assay revealed that the BMCs of siSTC1-SHED-HepTx mice, siSTC1-SHED-HepTx-BMCs, increased the number of TRAP-positive MNCs and expression of *Tnfrsf11a*, *Nfatc1*, and *Ctsk* compared to the BMCs of siCONT-SHED-HepTx mice, siCONT-SHED-HepTx-BMCs (Figure 7A, B and Supplementary Figure 12). The siSTC1-SHED-HepTx-BMCs showed lower expression of *Il-17* and *Tnfrsf11a* than the siCONT-SHED-HepTx-BMCs (Figure 7C). Further co-culture experiment of calvarial osteoblasts and Cont-BMCs demonstrated that the IL-17 supplement increased the number of TRAP-positive MNCs and expression of *Tnfrsf11a*, *Nfatc1*, and *Ctsk*; however, the treatment with anti-TNFSF11A antibody neutralized the IL-17-enhanced effects but did not affect the control IgG treatment (Figure 7D, E and Supplementary Figure 13).

ELISA demonstrated that the CCl_4_ mice expressed the increased serum levels of TGFB compared to the control mice (Supplementary Figure 14A), which is correlated with previous studies that reflect to the osteoblast dysfunction in bone loss of chronic liver disease [31,32]. siSTC1-SHED-HepTx attenuated the recovered serum levels of TGFB of siCONT-SHED-HepTx in the CCl_4_ mice (Supplementary Figure 14A). *In vitro* osteogenic capacity demonstrated that the CCl_4_-BMSCs exhibited the decreased osteogenic capacity compared to the Cont-BMSCs by Alizarin Red staining and RT-qPCR, but the BMSCs of siSTC1-SHED-HepTx mice, siSTC1-SHED-HepTx-BMSCs, suppressed the improved osteogenic capacity compared to the BMSCs of siCONT-SHED-HepTx mice, siCONT-SHED-HepTx-BMSCs (Supplementary Figure 14B–D). SEMA3A is known as an osteoprotective factor produced by osteoblasts and regulates bone volume by a balance between osteoclastogenic suppression and osteogenic promotion [33]. The CCl_4_-BMSCs exhibited the decreased expression of *Sema3a* compared to the Cont-BMSCs two weeks after osteogenic induction by RT-qPCR but the siSTC1-SHED-HepTx-BMSCs improved the *Sema 3a* level in the siCONT-SHED-HepTx-BMSCs (Supplementary Figure 14D).

## 4 Discussion

We demonstrate that hepatic fibro-inflammation is caused by hepatic ROS released from damaged hepatocytes in CCl_4_-induced chronic liver disease model mice. MSC-releasing STC1 play an important role in treating several ROS-induced diseases, including retinal degeneration, obesity-induced hepatitis, and lung fibrosis [[34], [35], [36]]. Recent transcriptomic and proteomic analyses reveal that the molecular mechanism of CCl_4_-damaged liver fibrosis is related to oxidative stress and PPAR signaling pathway [37]. A previous study demonstrates that SHED-Hep-secreting STC1 suppressed ROS-mediated hepatocyte necrosis in Wilson’s disease model rats [19]. The present siSTC1-SHED-HepTx showed the attenuation of therapeutic benefits of SHED-HepTx on abundant ROS production and fibro-inflammation in chronically CCl_4_-induced liver fibrosis. These findings suggest that hepatic ROS-targeting may offer a novel modality for treating chronic liver fibrosis in SHED-Hep-based therapy.

The present study demonstrates that CCl_4_ exhibits liver toxicity, but does not cause bone toxicity, indicating that the liver-releasing factors affects bone metabolism in CCl_4_-induced chronic liver disease, as correlated with the previous studies [32,38]. However, the regulation in the liver–bone axis was unknown. Recently, liver releasing factors including insulin-like growth factor binding protein 1, vitamin D, and TGFB participate in a liver–bone axis to cause the bone reduction in chronic liver disease [32,38]. It is known that IL-17 increases the expression of TNFSF11 on osteoblasts to induce osteoclast differentiation via TNFRSF11A [39]. Our *in viv*o and *in vitro* studies indicate that liver releasing ROS induced expression of *Il-17* and *Tnfrsf11a* in BMCs enhances the osteoclast formation via TNFSF11–TNFRSF11A signaling. Moreover, we show that hepatic ROS-induced osteoblast dysfunction is associated with the bone reduction in CCl_4_-induced mice, as reported previously in cholestasis of patients and bile duct ligated or CCl_4_-treated mice [31,32,40,41], suggesting that liver releasing ROS exacerbate the bone loss through the imbalance between osteoclast and osteoblast activities in CCl_4_-induced mice. Further functional knock-down of STC1 in donor SHED-Heps attenuate the suppressed osteoclast and inducible osteoblast functions of SHED-HepTx in CCl_4_-induced mice. Thus, these findings suggest that liver releasing ROS target the bone cells including BMCs and BMSCs to cause bone loss through the imbalance between osteoclast and osteoblast differentiation in chronic liver fibrosis and indicated that SHED-Hep-based therapy targets liver releasing ROS to regulate the bone metabolism, as well as fibro-inflammation, in chronic liver fibrosis.

We speculate another pathological sequence of gained expression of IL-17 in BMCs of CCl_4_ induced mice. Liver-releasing ROS recruits IL-17-producing immune cells into the injured liver of chronic liver disease [42,43]. Recent study shows that *Saa*-overexpression recruits IL-17-secreting neutrophils in bone marrow, leading to exacerbating bone loss [44]. An increased level of circulating Th17 cells is implicated in bone loss in primary sclerosing cholangitis [45]. Given the present results that CCl_4_-induced hepatic ROS enhances the expression of bone marrow *Il-17* and secretion of hepatic SAA1 in chronically CCl_4_-treated mice, we speculate that IL-17-secreting immune cells may contribute the liver–bone axis to induce bone loss in chronic liver diseases. Further study will be necessary to elucidate the mechanism of requiting IL-17-producing cells into bone marrow under hepatic ROS condition.

Given the present findings that liver-releasing ROS targets BMSCs in chronically CCl_4_-treated mice, we suppose another possibility of anti-bone loss efficacy in SHED-Hep-based therapy that the recipient BMSC-targeting STC1 released from SHED-Heps might contribute the bone recovery in chronic liver disease. Recent STC1 knock-in and knock-down study shows the STC1-enriched EVs released from adipose MSCs participate in angiogenesis in carotid endarterium mechanical injury [46]. SHED-releasing EVs play a crucial role in targeting the recipient bone marrow MSCs in osteoporotic and autoimmune model mice [22,23]. STC1 enhances the differentiation and bone formation of osteoblasts in an autocrine/paracrine manner [47]. Locally secreted STC1 is also known to act as a hormone to regulate distant tissues/organs [48,49]. We demonstrate that siSTC1-SHED-HepTx attenuate the improvement of the osteogenic and osteoclastogenic functions of the recipient BMSCs in chronically CCl_4_-treated mice with SHED-Hep-Tx. Further study will be necessary to elucidate the mechanism of SHED-Hep-releasing STC1 target the recipient BMSCs in chronic liver disease.

Taken together, the present findings suggest hepatic ROS-induced chronic liver fibrosis causes bone loss by the imbalance of osteoclast and osteoblast activities in a liver–bone axis. The present study also indicate that targeting of hepatic ROS may provide a valuable means for anti-bone loss treatment, as well as anti-fibro-inflammatory treatment, in chronic liver fibrosis. This hepatic ROS-targeting SHED-Hep-based approach may provide a feasible tool for the development of effective therapies for various liver disorders and their associated secondary disorders.

## Author contribution

Conceptualization, TY; Formal analysis, SS, SM, and TY; Investigation, SS, SM, HY, RY, JF, KY, TM, and TY; Resources, SS, HY, and TY; Data curation, SS, SM, HY, and TY; Writing – Original Draft, TY; Writing – Review & Editing, all authors; Visualization, SS and TY; Supervision, SO, TaT, ToT, and TY; Project administration, SS and TY; Funding acquisition, SS, SM, and TY; All authors approved the manuscript for publication.

## Data Availability

No data was used for the research described in the article.
