# Supplementary material for: Targeting hepatic oxidative stress rescues bone loss in liver fibrosis
Source: Mol Metab. 2022 Sep 13;66:101599. doi: 10.1016/j.molmet.2022.101599 (PMC9515604; doi:10.1016/j.molmet.2022.101599)
Supplement: Supplementary file 1 — Multimedia component 1 [file mmc1.pdf]

Supplementary Information

Targeting hepatic oxidative stress rescues bone loss in liver fibrosis

Soichiro Sonoda, Sara Murata, Haruyoshi Yamaza, Rathi Yuniartha, Junko Fujiyoshi, Koichiro Yoshimaru, Toshiharu Matsuura, Yoshinao Oda, Shouichi Ohga, Tasturo Tajiri, Tomoaki Taguchi, Takayoshi Yamaza

Supplementary Materials

Supplementary Table 1. A list of specific antibodies for flow cytometric analysis

**Antibodies, antigens**	**Conjugates, subclasses, hosts, clones**	**Sources**
anti-CD11b antibody, human	R-PE-conjugated IgG1 kappa, mouse, HI111	BioLegend
anti-CD14 antibody, human	R-PE-conjugated IgG1 kappa, mouse, 63D3	BioLegend
anti-CD19 antibody, human	R-PE-conjugated IgG1 kappa, mouse, 47G	BioLegend
anti-CD29 antibody, human	R-PE-conjugated IgG1 kappa, mouse, VA-S202	BioLegend
anti-CD34 antibody, human	R-PE-conjugated IgG2a kappa, mouse, 561	BioLegend
anti-CD45 antibody, human	R-PE-conjugated IgG1 kappa, mouse, 2D1	BioLegend
anti-CD90 antibody, human	R-PE-conjugated IgG1 kappa, mouse, 5E10	BioLegend
anti-CD73 antibody, human	R-PE-conjugated IgG1 kappa, mouse, AD2	BioLegend
anti-CD105 antibody, human	R-PE-conjugated IgG1 kappa, mouse, 43A3	BioLegend
anti-CD146 antibody, human	R-PE-conjugated IgG1 kappa, mouse, P1H112	BioLegend
anti-HLA-DR antibody, human	R-PE-conjugated IgG2a kappa, mouse, L243	BioLegend
mouse IgG1 kappa	R-PE-conjugated IgG1 kappa, mouse, MOPC-21	BioLegend
mouse IgG2a kappa	R-PE-conjugated IgG2a kappa, mouse, MOPC-173	BioLegend

HLA-DR: human leukocyte antigen DR; R-PE: R-phycoerythrin

Supplementary Table 2. A list of TaqMan probes for human genes.

Genes	Gene assay ID Numbers	Genes	Gene assay ID Numbers
ACAN	Hs00153936_m1	*NR1I2*	Hs00243666_m1
AFP	Hs00173490_m1	*PPARA*	Hs00231882_m1
ALB	Hs00910225_m1	*PPARG*	Hs0115513_m1
BGLAP	Hs01587814_g1	*RUNX2*	Hs00231692_m1
HNF1A	Hs00167041_m1	*SOX9*	Hs01001343_g1
HNF4A	Hs00230853_m1	*STC1*	Hs00174970_m1
KRT18	Hs02827483_g1	*TTR*	Hs0174914_m1
LPL	Hs00173425_m1	18S rRNA	Hs99999901_s1

ACAN, aggrecan; AFP, alpha fetoprotein; ALB, albumin; BGLAP, bone gamma-carboxyglutamate protein; CPS1, carbamoyl-phosphate synthase 1; HNF1A, hepatocyte nuclear factor 1A; HNF4A, hepatocyte nuclear factor 4A; LPL, lipoprotein lipase; NR1I2, nuclear receptor subfamily 1 group I member 2; PPARA, peroxisome proliferator activated receptor alpha; PPARG, peroxisome proliferator-activated receptor gamma; RUNX2, runt related transcription factor 2; SOX9, SRY-box9; STC1, stanniocalcin 1; TTR, transthyretin.

Supplementary Table 3. The list of TaqMan probes for mouse genes.

Genes	Gene assay ID Numbers
Acta2	Mm00725412_s1
Alp	Mm00475834_m1
Bglap	Mm03413826_mH
Col1a1	Mm00801666_g1
Ctsk	Mm000484039_m1
IL17	Mm00439618_m1
Nfatc1	Mm01265944_m1
Nox4	Mm00479246_m1
Pparg	Mm00440940_m1
Runx2	Mm00501584_m1
Saa1	Mm99999915_g1
Sema3a	Mm00436469_m1
Tnfrsf11a	Mm00437135_m1
18S rRNA	Mm03928990_g1

Acta2, actin, alpha 2, smooth muscle; Alp, alkaline phosphatase; Bglap, bone gamma carboxyglutamate protein; Col1a1, collagen, type I, alpha 1; Ctsk, cathepsin k; Il17, interleukin 17; Nftac1, nuclear factor of activated T cells 1; Nox4, NADPH oxidase 4; Pparg, peroxisome proliferator-activated receptor gamma; Runx2, runt related transcription factor 2; Saa1, serum amyloid A1; Sema3a, semaphoring 3a; Tnfrs11a, tumor necrosis factor receptor superfamily, member 11a.

Supplementary Table 4. The list of commercially available kits for colorimetric and enzyme labelled immunosorbent assays.

Tests	Kits	Sources
ALB	AssayMAX Human Albumin ELISA Kit	AssayPro
ALT, AST	Transaminase CII-Test Kit	FUJIFILM Wako Pure Chemicals
Bilirubin, total	QuantiChrom Bilirubin Assay Kit	BioAssay Systems
CTX-I	Collagen I ELISA kit, RatLaps	Nordic Bioscience Diagnostics A/S
Glucose	Glucose CII-test	FUJIFILM Wako Pure Chemicals
GSH-PX	Glutathione peroxidase assay kit	Nikken Seil
HYP	Hydroxyproline Assay Kit	Biovision
MDA	TBARS assay kit	Cayman Chemical
SAA	Mouse SAA ELISA Kit	Abcam
TGFB	Mouse TGF-β Quantikine ELISA kit	R&D Systems
TRAP5b	TRAcP 5b ELISA kit, RatLaps	Nordic Bioscience Diagnostics A/S
Triglyceride	Triglyceride E-test 1	FUJIFILM Wako Pure Chemicals
Urea	Urea Assay Kit	Abcam

ALB, albumin; ALT, alanine aminotransferase; AST, aspartate aminotransferase; CTX-I: C-terminal telopeptide of type I collagen; GSH-PX, glutathione peroxidase; HYP, Hydroxyproline; MDA, malondialdehyde; SAA1, serum amyloid A1; TGFB, transforming growth factor beta; TRAP5b, tartrate-resistant acid phosphatase 5b.

Supplementary Table 5. The list of specific antibodies for immunohistochemistry and immunofluorescence.

**Antibodies, antigens**	**Subclasses, hosts, clones**	**Sources**
anti-ACTA2 antibody, mouse	purified IgG2a, mouse, ASM-1/1A4	Merck
anti-HepPar1 antibody, human	purified IgG1, mouse, OCH1E5	Abcam
anti-HLA-ABC antibody, human	purified IgG2a, mouse, W6/32	Abcam
mouse IgG1 kappa	purified IgG1 kappa, mouse, MOPC-21	Abcam
mouse IgG2a kappa	purified IgG2a kappa, mouse, MOPC-173	Abcam

ACTA2, actin, alpha 2, smooth muscle; HepPar1, human hepatocyte paraffin 1; HLA-ABC, human leukocyte antigens A, B, and C.

Supplementary Methods

Isolation and culture of stem cells from human exfoliated deciduous teeth (SHED)

SHED were isolated from remnant dental pulp tissues of human exfoliated deciduous teeth by a colony-forming unit-fibroblast (CFU-F) method [1,2]. The tooth samples were collected as discarded clinical samples from healthy pediatric donors (6–7 years old, n = 3). The dental pulp tissues were extracted from the teeth and digested with 0.3% collagenase type I (Worthington Biochemicals, Lakewood, NJ, USA) and 0.4% dispase II (Sanko Junyaku, Tokyo, Japan) for 60 min at 37°C. The cell suspension was seeded on T75-flasks (Corning, Corning, NY, USA). Non-adherent cells were removed by washing with sterilized phosphate-buffered saline (PBS) 24 hours after seeding. The adherent cells were maintained in a complete growth medium (CGM) to form attached colonies on the flasks. The adherent colony-forming cells were passaged, and the passaged 3 (P3) cells were used for further experiments. The CGM consisted of 15% fetal bovine serum (FBS; Equitech-Bio, Kerrville, TX, USA), 100 μM L-ascorbic acid 2-phosphate (Fuji Film Wako Chemicals, Osaka, Japan), 2 mM L-glutamine (Nacalai Tesque, Kyoto, Japan), and

premixed antibiotics containing 100 U/mL penicillin and 100 μg/mL streptomycin (premixed P/S; Nacalai Tesque) in alpha Modification of Eagle's Medium (αMEM; Thermo Fisher Scientific, Waltham, MA, USA). The medium was changed twice a week. The cells were determined the characteristics as mesenchymal stem cells as described previously [3].

Generation of SHED-converted hepatocyte-like cells, SHED-Heps

P3 SHED were seeded on human fibronectin-coated 100-mm culture dishes (Corning, Corning) and maintained confluently under the CGM. They were cultured under a hepatogenic inductive condition as reported previously [4]. They were maintained under serum-free Iscove's modified Dulbecco's media (Nacalai Tesque) and premixed P/S (Nacalai Tesque) supplemented with hepatogenic cytokines and regents. They were initially incubated with epidermal growth factor (EGF; 20 ng/mL; PeproTech, Cranbury, NJ, USA) and fibroblast growth factor 2 (FGF2; 10 ng/mL; PeproTech) for 2 days and subsequently stimulated with FGF2 (10 ng/mL; PeproTech), hepatocyte growth factor (HGF; 20 ng/mL; PeproTech) and nicotinamide (0.61 g/L; Merck, Darmstadt, Germany)

for 7 days. Finally, the cells were stimulated with oncostatin M (20 ng/mL; PeproTech), dexamethasone (1 μM; Merck), and ITS premix (1×; Corning) for 21 days. The mediums were changed twice weekly throughout the hepatogenic culture.

Treatment of small interfering RNA (siRNA)

SHED-Heps were pretreated for 3 days with siRNA for *stanniocalcin 1* (*STC1*) and its scrambled control, referred to as siSTC1 and siCONT (20 nM; Santa Cruz Biotechnology, Santa Cruz, CA, USA) in αMEM (Thermo Fisher Scientific) without antibiotics using a transfection reagent Lipofectamine RNAiMAX (Thermo Fisher Scientific). The siRNA treated cells was analyzed the gene and protein expression of STC1 in SHED-Heps and STC1 secretion in conditioned medium by quantitative reverse transcription polymerase chain reaction (RT-qPCR), immunofluorescent analysis, and enzyme-linked immunosorbent assay (ELISA).

In vitro osteoclast differentiation assay

Calvarial osteoblasts were isolated from parietal bones of newborn C57BL/6J (1–3 days old) by a sequential enzyme treatment [5]. Briefly, the newborn parietal bones were sequentially treated with 3 mg/mL collagenase type I (Worthington Biochemicals) and 4 mg/ml dispase (Fuji Film Wako Chemicals) and cultured on 100 mm dishes with a complete medium consisting of 10% FBS (Equitech-Bio), 2 mM L-glutamine (Nacalai Tesque), and premixed P/S (Nacalai Tesque) in αMEM (Thermo Fisher Scientific). Calvarial osteoblasts were passed and seeded at 10×10^3 per well on 24-well multiplates (Corninig). Bone marrow cells (BMCs; 200×10^3 per well) were obtained from mouse femurs and tibiae and cocultured with calvarial osteoblasts in 10% FBS (Equitech-Bio), 1 alpha, 25 dihydroxyvitamin D_3 (10 nM; FUJIFILM Wako Chemicals), and prostaglandin E2 (1 nM; FUJIFILM Wako Chemicals), and premixed P/S (Nacalai Tesque) in αMEM (Thermo Fisher Scientific) for 7 days, as described before [6]. The half medium was changed every 3 days. Some co-cultures were treated with recombinant interleukin 17 (10 nM; PeproTech) and/or anti-tumor necrosis factor superfamily 11 (50 mg/mL; R&D Systems, Minneapolis, MN, USA) or goat IgG (50 ng/mL; R&D Systems). The co-cultures were analyzed by tartrate-resistant acid phosphatase (TRAP) staining at

37°C by using an acid phosphatase, Leukocyte (TRAP) kit (Merck) according to the manufacturer's instructions. TRAP-positive multinucleated cells (>3 nuclei) were counted as osteoclast-like cells under a light microscopy.

In vitro osteoblast inductive assay

Mouse bone marrow stromal cells (BMSCs) were isolated from femurs and tibias of mice and expanded in 10% FBS (Equitech-Bio), 2 mM L-glutamine (Nacalai Tesque), and premixed P/S (Nacalai Tesque) in αMEM (Thermo Fisher Scientific) according to the previous study [5]. Mouse BMSCs were cultured under an osteogenic condition with 50 μg/ml ascorbic acid, 5 mM β-glycerophosphate, and 10 nM dexamethasone. Mineralized nodule formation was determined four weeks after osteogenic condition using 1% Alizarin Red solution (Merck) and the positive area was measured using an ImageJ (National Institutions of Health, Bethesda, MD) [5]. The expression semaphoring 3a (*Sema3a*) was also analyzed using RT-qPCR.

Flowcytometric (FCM) analysis

Cultured cells (0.1 × 10^6) were suspended in 100 μL of Hanks' balanced salt solution (HBSS; Nacalai Tasque) at 4°C. The cell suspension was incubated with R-phycoerythrin (R-PE) conjugated primary antibodies (1 μg per 100 μL FCM buffer, **Supplementary Table 1**) at 4°C for 45 min and washed at 4°C in a FCM buffer. The FCM buffer consisted of 2% heat inactivated FBS (Merck) in Hanks' balanced salt solution (HBSS). The immunostained cells were measured on FACSVerse flow cytometer (BD Biosciences, Franklin Lake, NJ, USA). As controls, isotype-matched antibodies conjugated with R-PE were used instead of the primary antibodies. The percentage of positive cells was determined using FACSuite software (BD Biosciences) compared control cells stained with corresponding isotype-matched antibodies in which a false-positive rate of less than 1% was accepted [6].

RT-qPCR assay

RNA samples were extracted from cell and tissue samples using a TRIzol regent (Thermo Fisher Scientific) and digested with DNase I (Promega, Madison, WI, USA). The total RNA was purified using a RNeasy Mini Kit (Qiagen, Venlo, Netherlands). The

complementary DNA (cDNA) was prepared from the purified total RNA by reverse transcription reaction using a Revertra Ace qPCR kit (TOYOBO, Tokyo, Japan) according to the manufacturer's instructions. Gene expression was analyzed in the cDNA mixed with EagleTaq Master Mix (Roche Applied Science, Babaria, Germany) and target TaqMan probes (Thermo Fisher Scientific, **Supplementary Tables 2 and 3**) with Light Cycler 96 system (Roche Applied Science). The PCR steps were as follows: preincubation 1 (50°C for 120 sec), preincubation 2 (95°C for 600 sec), and two step amplification (95°C for 15 sec and 60°C for 60 sec at 45 cycles). Human and mouse 18S ribosomal RNAs were used for normalization.

ELISA and colorimetric assays

Total protein concentration of samples was quantified using Bio-Rad protein assay (Bio-Rad Laboratories, Hercules, CA) to ensure equal loading of each well. ELISA and colorimetric assay were performed using commercially available kits (**Supplementary Table 4**) according to the manufacturers' instructions. The results were measured with Multiskan GO (Thermo Fisher Scientific).

Immunohistochemical and double immunofluorescent analyses

Mouse livers and tibiae were fixed with 4% paraformaldehyde in PBS and processed for paraffin embedding. Some paraffin sections were incubated with 3% hydrogen peroxide in methanol for 30 min and treated with 5% normal goat serum (Thermo Fisher Scientific) in PBS. They were incubated with primary antibodies overnight at 4°C, followed by treating with Dako Envision+ system-HRP labeled polymer anti-rabbit or anti-mouse (Agilent, Santa Clara, CA, USA). The sections were visualized with 0.05% diaminobenzidine-4HCl (Dojindo Laboratories, Kumamoto, Japan) and 0.006% hydrogen peroxide for 5 min and counter-stained with hematoxylin.

For double immunofluorescence, the other paraffin sections were blocked with 5% normal donkey serum (Thermo Fisher Scientific) and incubated with the first primary antibody, followed by treating with Alexa Fluor 488-conjugated secondary antibodies (Thermo Fisher Scientific). Then they were treated with the second primary antibody, followed by treating with Alexa Fluor 568-conjugated secondary antibodies (Thermo Fisher Scientific). All immunofluorescent samples were stained with 4', 6-diamidino-2-

phenylindole (1 μg/mL; Thermo Fisher Scientific). The target specific antibodies are summarized in **Supplementary Table 5**. Immunohistochemical controls were performed with non-immune mouse IgG_1 and mouse IgG_{2a} instead of the primary antibodies. All sections were observed under Axio Imager microscopy (Carl Zeiss Microscopy, Jena, Germany) equipped with Apoptome.2 optical sectioning system (Carl Zeiss Microscopy).

References

[1] Friedenstein, A.J., Deriglasova, U.F., Kulagina, N.N., Panasuk, A.F., Rudakowa, S.F., Luriá, E.A., et al. 1974. Precursors for fibroblasts in different populations of hematopoietic cells as detected by the in vitro colony assay method. Experimental Hematology 2:83–92.

[2] Yamaza, T., Kentaro, A., Chen, C., Liu, Y., Shi, Y., Gronthos, S., et al. 2010. Immunomodulatory properties of stem cells from human exfoliated deciduous teeth. Stem Cell Research & Therapy 1:5. Doi: 10.1186/scrt5.

[3] Iwanaka, T., Yamaza, T., Sonoda, S., Yoshimaru, K., Matsuura, T., Yamaza, H., et al. 2020. A model study for the manufacture and validation of clinical-grade

deciduous dental pulp stem cells for chronic liver fibrosis treatment. Stem Cell Research & Therapy 11:134. Doi: 10.1186/s13287-020-01630-w.

[4] Fujiyoshi, J., Yamaza, H., Sonoda, S., Yuniartha, R., Ihara, K., Nonaka, K., et al. 2019. Therapeutic potential of hepatocyte-like-cells converted from stem cells from human exfoliated deciduous teeth in fulminant Wilson's disease. Scientific Reports 9:1535. Doi: 10.1038/s41598-018-38275-y.

[5] Danjo, A., Yamaza, T., Kido, M.A., Shimohira, D., Tsukuba, T., Kagiya, T., et al., 2007. Cystatin C stimulates the differentiation of mouse osteoblastic cells and bone formation. Biochemical and Biophysical Research Communications 360:199–204. Doi: 10.1016/j.bbrc.2007.06.028.

[6] Sonoda, S., Murata, S., Nishida, K., Kato, H., Uehara, N., Kyumoto, Y.N., et al. 2020. Extracellular vesicles from deciduous pulp stem cells recover bone loss by regulating telomerase activity in an osteoporosis mouse model. Stem Cell Research & Therapy 11:296. Doi: 10.1186/s13287-020-01818-0.

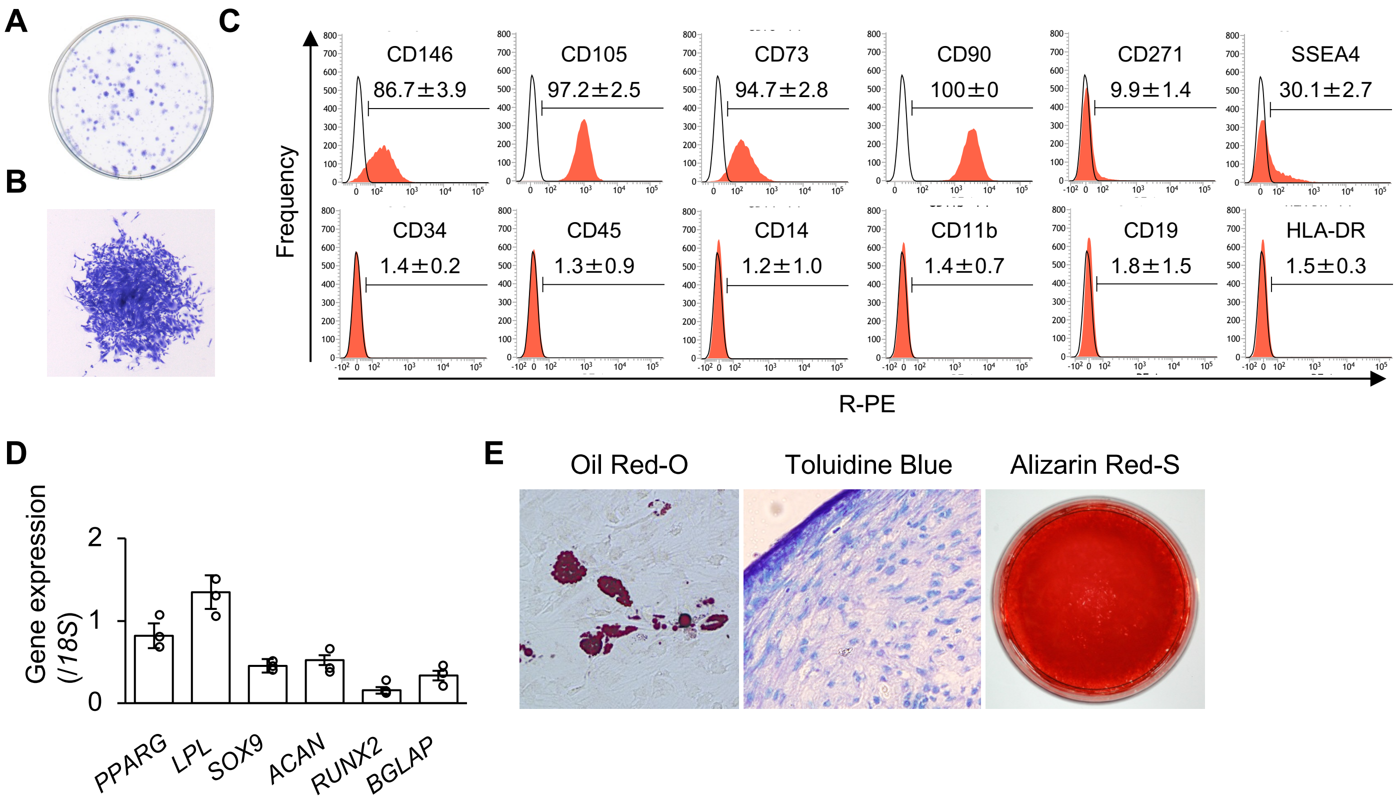

Supplementary Figure 1. Characterization of stem cells from human exfoliated deciduous teeth (SHED). (**A, B**) Representative images of SHED show attached colony formation on a culture dish (**A**) and a spindle-shaped cell cluster (**B**) by toluidine blue staining. (**C**) Representative histograms show the expression of cell surface markers on SHED by flow cytometric analysis. Areas filled with red; target antibody-stained histograms; solid lines; isotype-matched control-stained histograms. SSEA4, stage specific embryonic antigen 4; HLA-DR, human leukocyte antigen DR; R-PE, R-phycoerythrin. n = 3. The numbers show the means ± standard error of mean (SEM) of positive rate. (**D, E**) Multipotency of SHED. The graph shows the expression of lineage specific genes for adipocytes, chondrocytes, and osteoblasts, 6, 4, and 1 weeks after adipogenic, chondrogenic, and osteogenic induction, respectively, by reverse transcription quantitative polymerase chain reaction (RT-qPCR). Results were shown as a ratio of *peroxisome proliferator activated receptor gamma 2* (*PPARG2*), *lipoprotein lipase* (*LPL*), *SRY-box 9* (*SOX9*), aggrecan (*ACAN*), *runt-related transcription factor 2* (*RUNX2*), and *bone gamma-carboxyglutamine protein* (*BGLAP*) to 18S ribosomal RNA (*/18S*). n = 3. The graph bars represent the means ± SEM (**D**). Representative images show lipid deposition, cartilage matrix formation, and mineralized tissue deposition 6, 4, and 5 weeks after adipogenic, chondrogenic, and osteogenic induction by Oil Red-O, toluidine blue, and Alizarin Red-S staining, respectively (**E**).

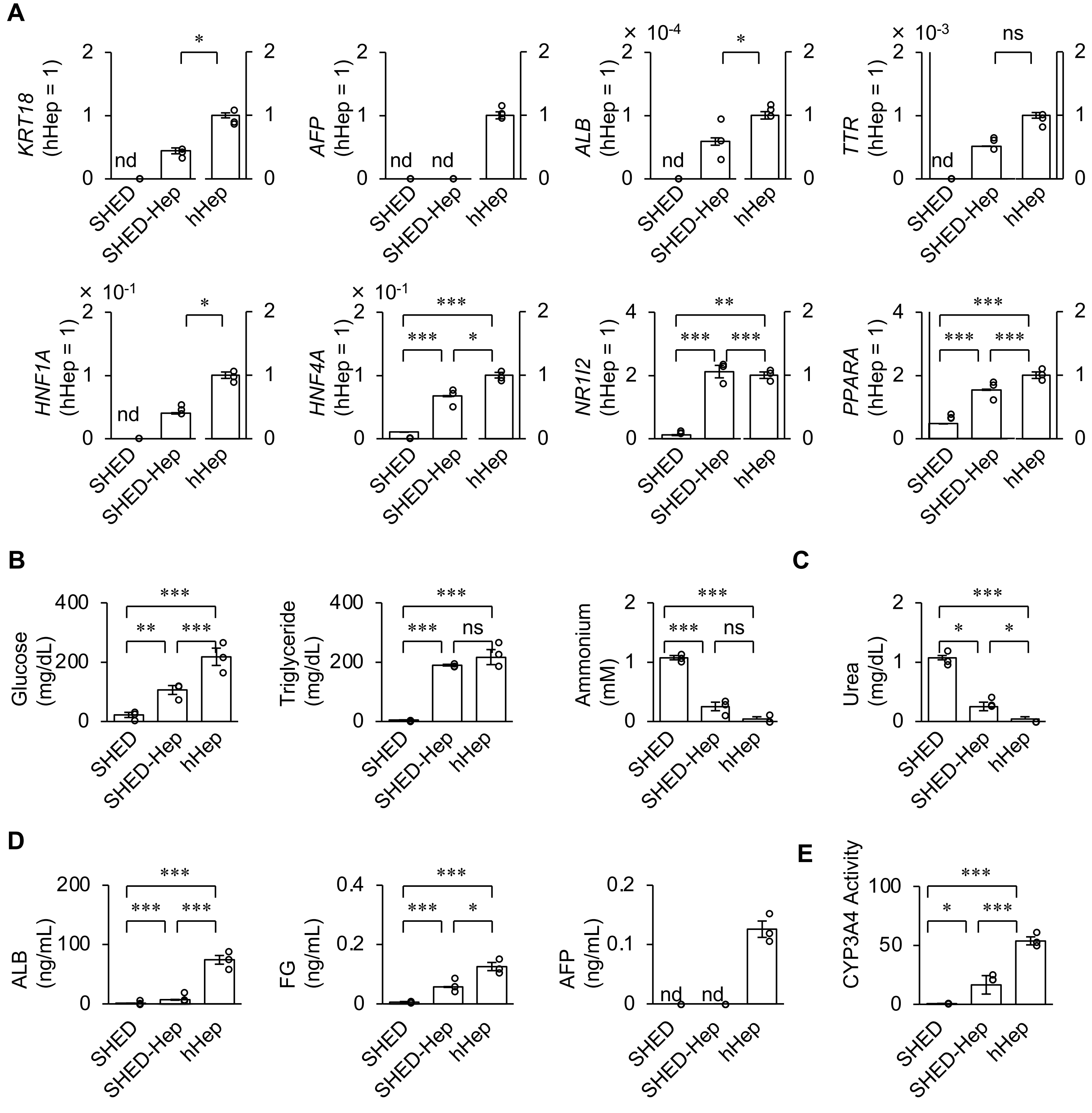

Supplementary Figure 2. Hepatogenic potency of SHED into immature hepatocyte-like cells, SHED-Heps. (**A**) The graphs show gene expression of albumin (*ALB*), alpha fetoprotein (*AFP*), keratin 18 (*KRT18*), hepatocyte nuclear factor 1A (*HNF1A*), *HNF4A,* nuclear receptor subfamily 1 group I member 2 (*NR1I2*), peroxisome proliferator activated receptor alpha (*PPARA*), and transthyretin (*TTR*) by RT-qPCR. Results are shown as ratios to human primary hepatocytes (hHep = 1). (**B**) The graphs show the amount of glucose, triglyceride, and ammonium in the conditioned medium by colorimetry assay. (**C**) The graphs show the intracellular amount of urea by colorimetry assay. (**D**) The graph shows the amount of ALB, fibrinogen (FG), and AFP in the CM by enzyme-labeled immunosorbent assays (ELISA). (**E**) The graph shows xenobiotic activity of cytochrome P450 3 subfamily A member 4 (CYP3A4) under dexamethasone loading. Results are shown as ratios to untreated cells in each culture condition. **A–E**: n = 3 per group. *$P < 0.05$; **$P < 0.01$; ***$P < 0.005$. nd, no detection; ns, no significance. The graph bars represent the means ± SEM.

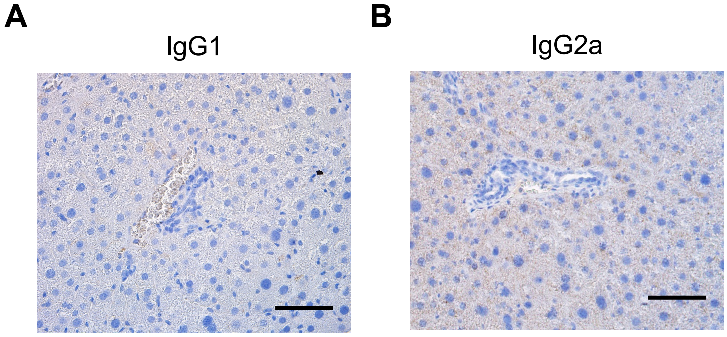

Supplementary Figure 3. Immunohistochemical control tests. (**A, B**) Representative images of control mouse livers are shown by immunohistochemical control tests with using isotype-matched immunoglobulins, including mouse IgG1 (**A**) and mouse IgG2a (**B**), instead of the primary antibodies. Sections were counterstained with hematoxylin. Scale bars, 50 μm.

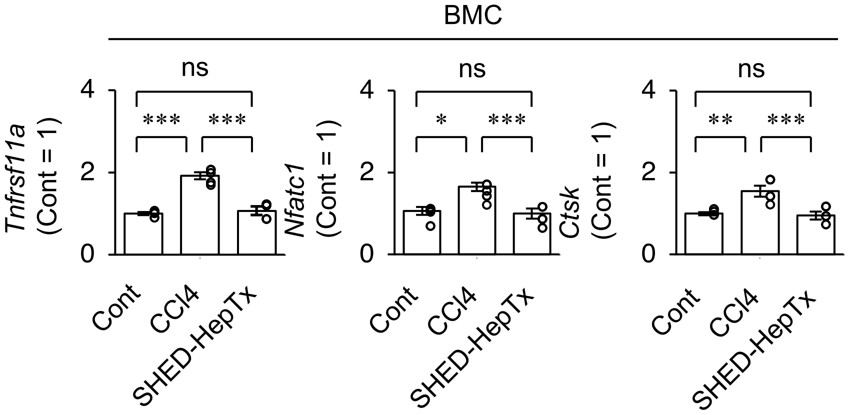

Supplementary Figure 4. Systemic SHED-Hep transplantation (SHED-HepTx) suppressed *in vitro* osteoclast differentiation of bone marrow cells (BMCs) in chronically CCl_4-treated mice. CCl_4-treated mice were harvested 4 weeks after SHED-HepTx. Mouse BMCs were co-cultured with calvarial osteoblasts in the presence of vitamin D_3 (VD_3; 10 nM) and prostaglandin E_2 (PGE_2; 1 nM). The expression of osteoclast markers, including *tumor necrosis factor receptor superfamily 11A* (*Tnfrsf11a*) , *nuclear factor of activated T-cell* (*Nfatc1*), and *cathepsin K* (*Ctsk*), by RT-qPCR. Results are shown as ratios compared to the co-cultures with BMCs derived from CCl_4-non-treated control mice (Cont = 1). Cont, olive oil-treated group; CCl4, CCl_4-treated group; SHED-HepTx, SHED-HepTx group. n = 10 for all groups. $*P < 0.05$, $**P < 0.01$, $***P < 0.005$. ns, no significance. The graph bars represent the mean ± SEM.

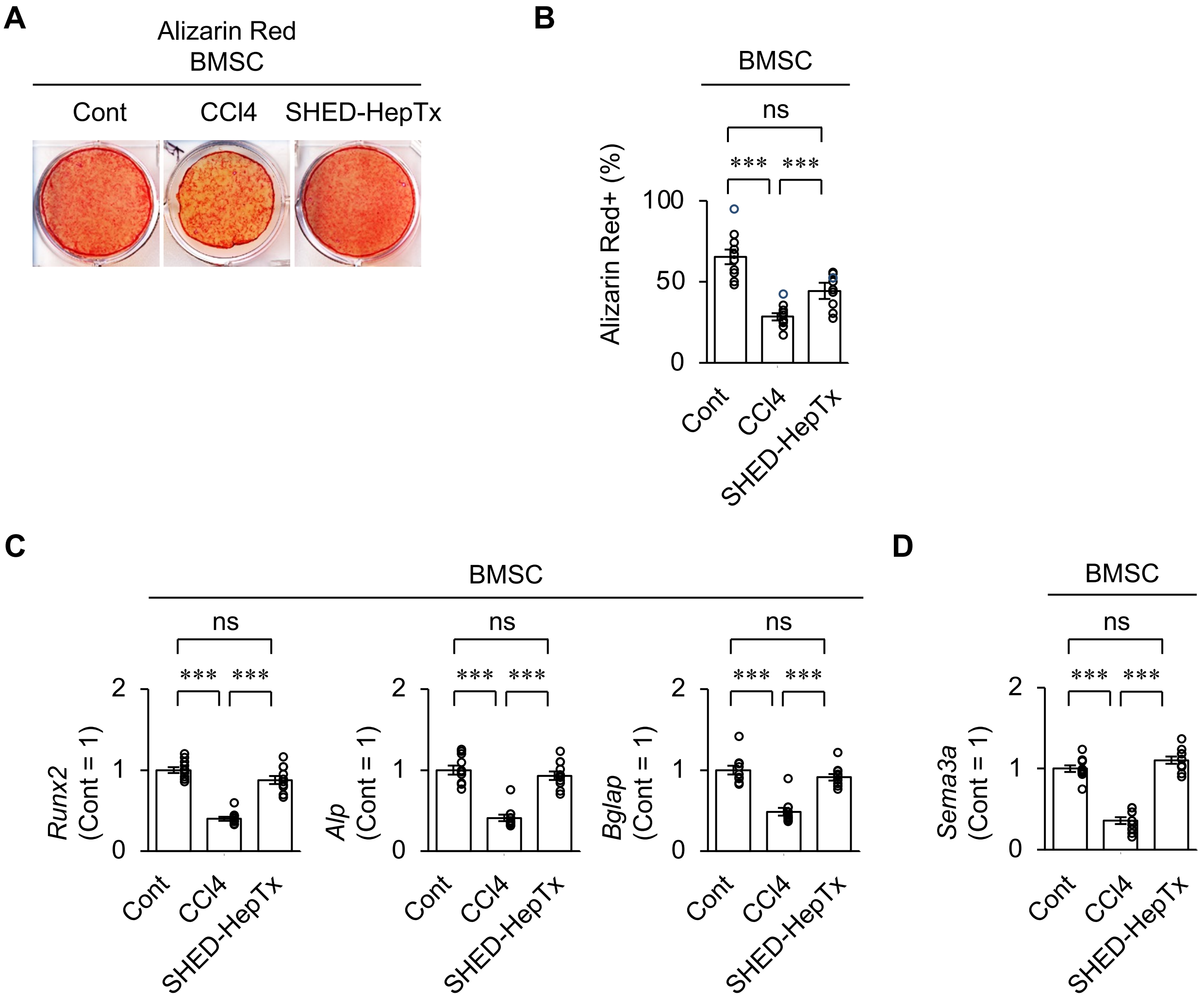

Supplementary Figure 5. Systemic SHED-HepTx recovered *in vitro* osteogenic function of bone marrow stromal cells (BMSCs) in chronically CCl_4-treated mice. CCl_4-treated mice were harvested 4 weeks after SHED-HepTx. Mouse BMSCs were cultured under an osteogenic induction with ascorbic acid (AA, 50 μg/mL), β-glycerophosphate (β-GP, 5 mM), and dexamethasone (Dex,10 nM) in the absence and presence of CCl_4 (2 μg/mL). (**A, B**) BMSCs were induced for 4 weeks under the osteogenic induction condition. Representative images of mineralized nodule formation were analyzed using Alizarin Red staining (**A**). The graph indicates the ratio of Alizarin Red-positive (Alizarin Red$^+$) area to total area (**B**). (**C, D**) BMSCs were induced for 2 weeks under the osteogenic induction condition analyzed by RT-qPCR. The graphs indicate the expression of osteoblast markers, *Runx2*, *alkaline phosphatase* (*Alp*), and *Bglap*, (**C**) and show the expression of semaphoring 3a (*Sema3a*) (**D**) in BMSCs. The results are presented as a ratio to the expression in BMSCs derived from CCl_4-non-treated control mice (Cont= 1). **A–D**: Cont, olive oil-treated group; CCl4, CCl_4-treated group; SHED-HepTx, SHED-HepTx group. **B–D**: n = 10 for all groups. ***P < 0.005. ns, no significance. The graph bars represent the mean ± SEM.

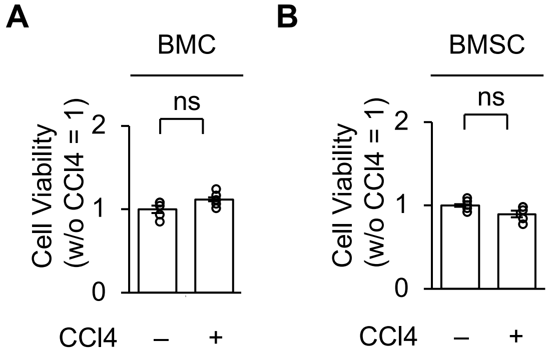

Supplementary Figure 6. Effects of CCl_4 on the cell survival of primary mouse BMCs and bone marrow stromal cells (BMSCs). (**A, B**) Mouse BMCs and BMSCs were stimulated with or without CCl_4 (2 μg/mL) for 4 days. The graph presents the cell viability in BMCs and BMSCs by colorimetric analysis. The results are presented as a ratio of the expression in BMCs and BMSCs without CCl_4 stimulation (w/o CCl4 = 1). n = 5 for all groups. ns, no significance. The graph bars represent the mean ± SEM.

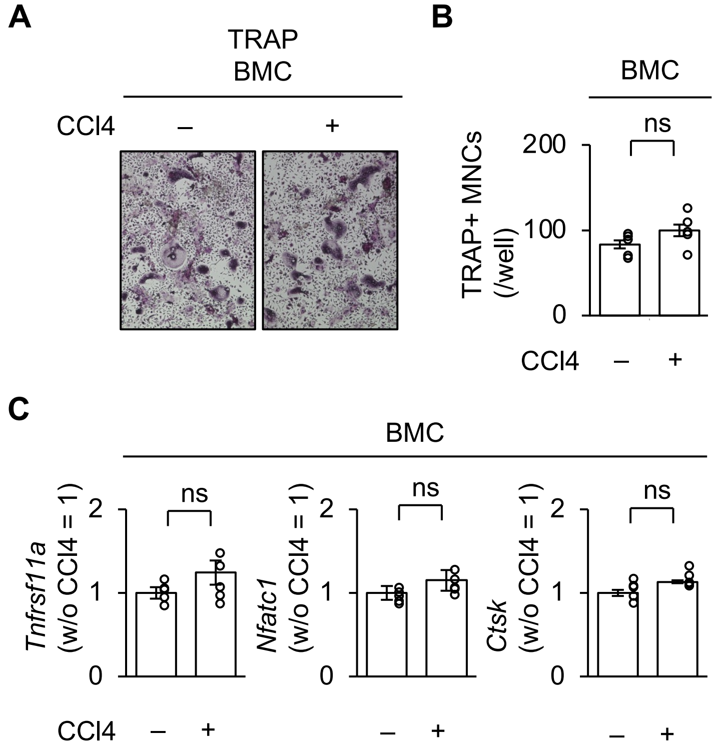

Supplementary Figure 7. Effects of CCl_4 on the *in vitro* osteoclast differentiation of BMCs. (**A–C**) Mouse BMCs were co-cultured with calvarial osteoblasts in the presence of VD_3 (10 nM) and PGE_2 (1 nM) in the absence and presence of CCl_4 (2 μg/mL). The presentative images of osteoclast differentiation were analyzed using tartrate resistant acid phosphatase (TRAP) staining (**A**). The graph indicates the number of TRAP-positive ($TRAP^+$) multinuclear cells (MNCs) (**B**). The expression of osteoclast markers, including *Tnfrsf11a*, *Nfatc1*, and *Ctsk* by RT-qPCR. Results are shown as ratios compared to the co-cultures with BMCs without CCl_4 stimulation (w/o CCl4 = 1) (**C**). **B, C**: n = 5 for all groups. ns, no significance. The graph bars represent the mean ± SEM.

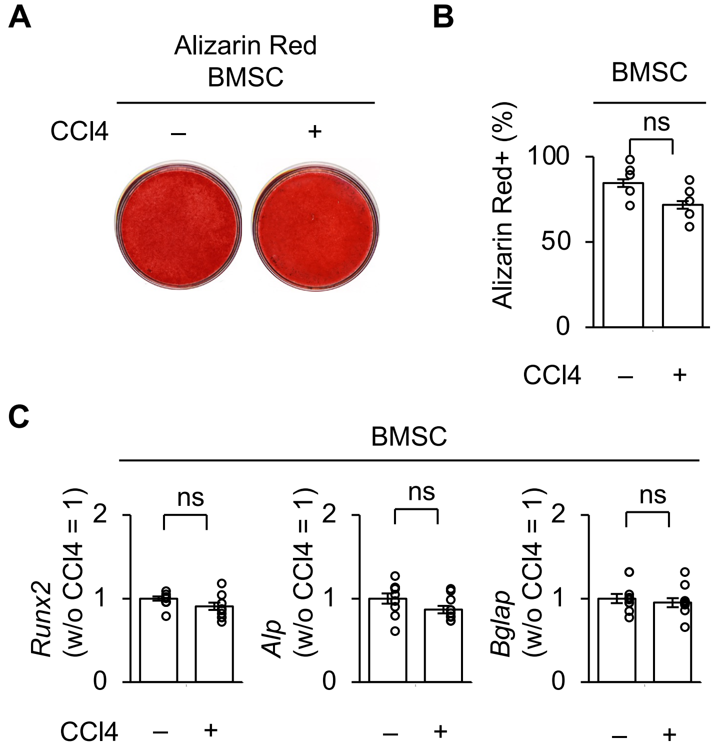

Supplementary Figure 8. Effects of CCl4 on the cell survival and *in vitro* bone formation of mouse BMSCs. Mouse BMSCs were cultured under an osteogenic induction with AA (50 μg/mL), β-GP (5 mM), and Dex (10 nM) in the absence and presence of CCl_4 (2 μg/mL). (**A, B**) BMSCs were induced for 4 weeks under the osteogenic condition. Representative images of mineralized nodule formation were analyzed using Alizarin Red staining (**A**). The graph indicates the ratio of Alizarin Red-positive (Alizarin Red^+) area to total area (**B**). (**C**) BMSCs were induced for 2 weeks under the osteogenic induction condition analyzed by RT-qPCR. The graphs indicate the expression of *Runx2*, *Alp*, and *Bglap* in BMSCs. The results are presented as a ratio to the expression in BMSCs without CCl_4 stimulation (w/o CCl4 = 1). **B, C**: n = 5 for all groups. ns, no significance. The graph bars represent the mean ± SEM.

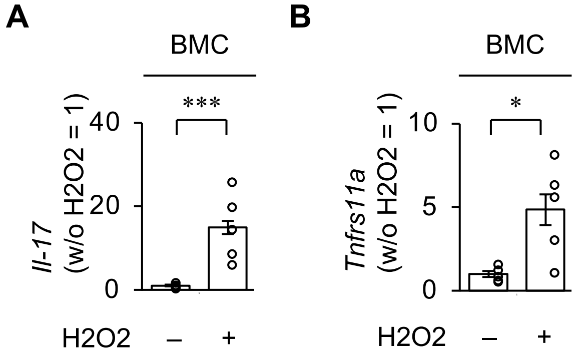

Supplementary Figure 9. Effects of H_2O_2 on the expression of *Il-17* and *Tnfs11a* in primary mouse BMCs. (**A, B**) Mouse BMCs were stimulated with or without H_2O_2 (0.1 mM) for 4 days. The graph presents the expression of *interleukin 17* (*Il-17*) and *Tnfrsf11a* in BMCs by RT-qPCR. The results are presented as a ratio of the expression in BMCs without H_2O_2 stimulation (w/o H2O2 = 1). n = 5 for all groups. $*P < 0.05$, $***P < 0.005$. The graph bars represent the mean ± SEM

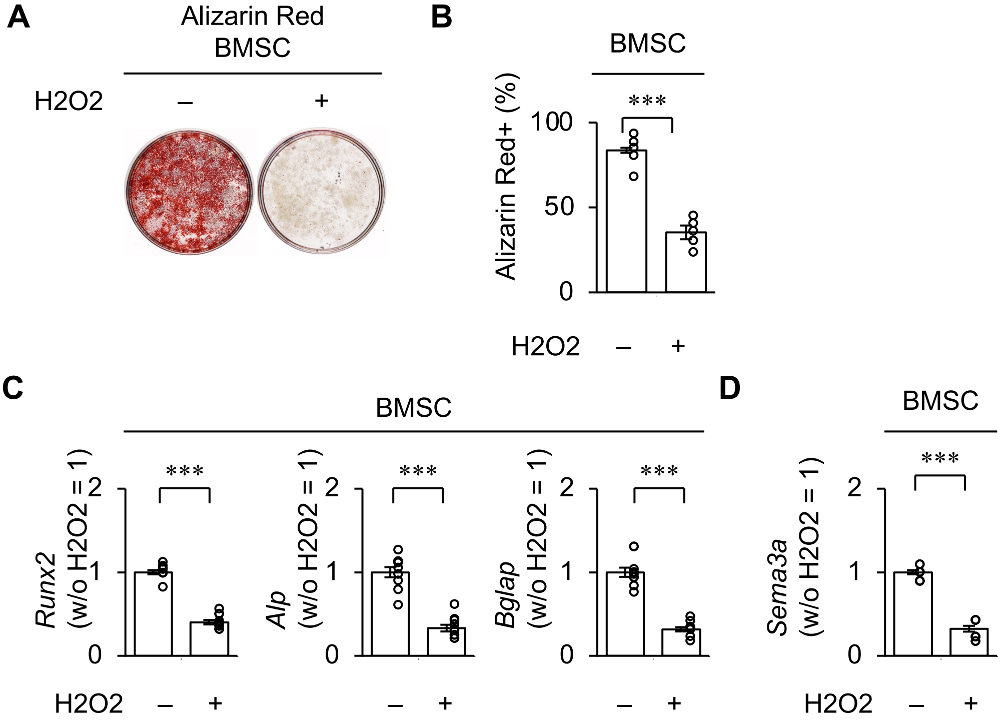

Supplementary Figure 10. Effects of H_2O_2 on the *in vitro* bone formation of mouse BMSCs. Mouse BMSCs were cultured under an osteogenic induction with AA (50 µg/mL), β-GP, (5 mM), and Dex (10 nM) in the absence and presence of H_2O_2 (0.1 mM). (**A, B**) BMSCs were induced for 4 weeks under the osteogenic condition. Representative images of mineralized nodule formation were analyzed using Alizarin Red staining (**A**). The graph indicates the ratio of Alizarin Red-positive (Alizarin Red$^+$) area to total area (**B**). (**C, D**) BMSCs were induced for 2 weeks under the osteogenic induction condition analyzed by RT-qPCR. The graphs indicate the expression of *Runx2*, *Alp*, and *Bglap* (**C**) and show the expression of *Sema3a* (**D**) in BMSCs. **B–D**: n = 5 for all groups. $***P < 0.005$. The graph bars represent the mean ± SEM. **C, D**: The results are presented as a ratio to the expression in BMSCs without H_2O_2 stimulation (w/o H2O2 = 1).

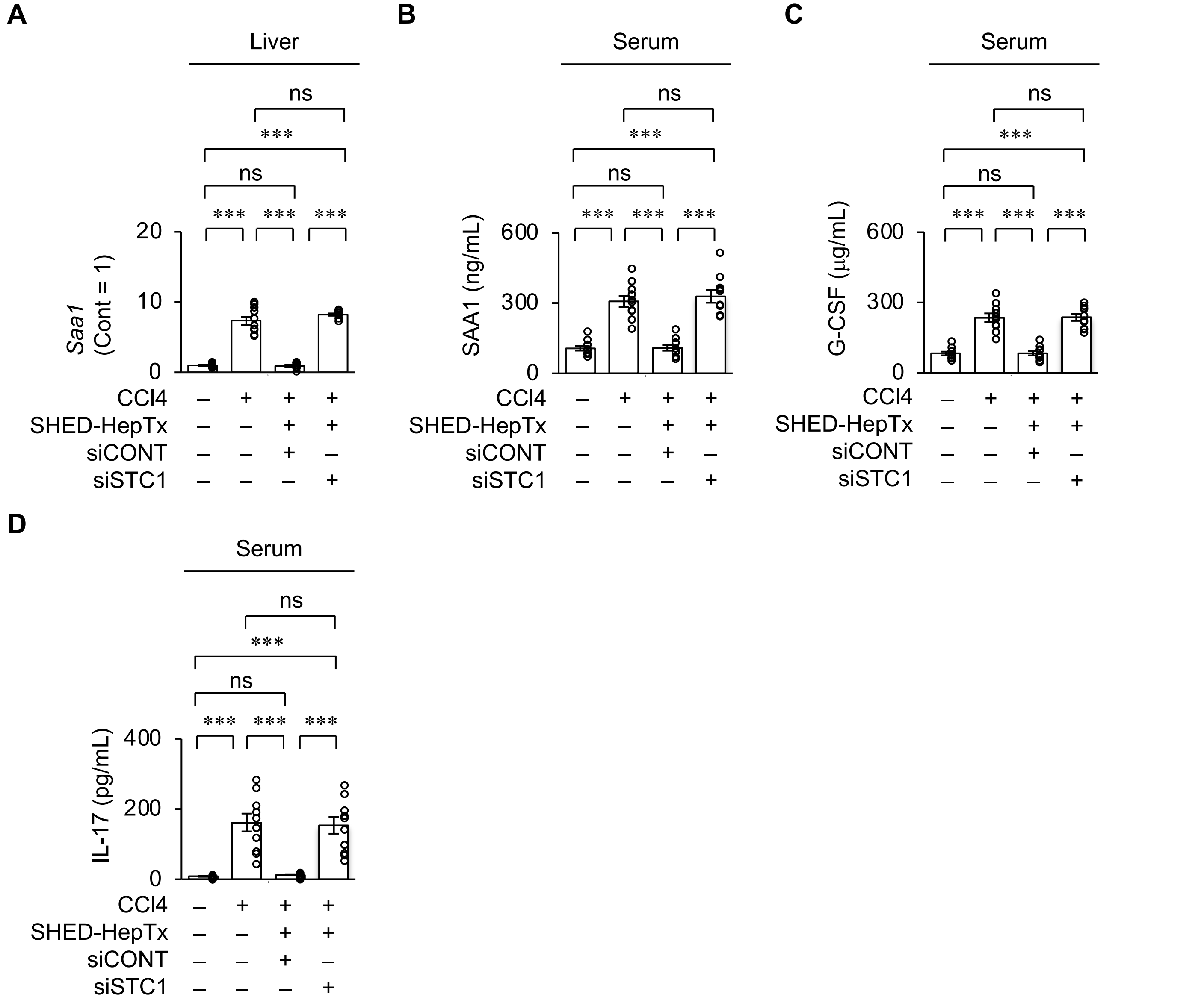

Supplementary Figure 11. STC1 knock-down attenuates the suppression of hepatic *serum amyloid A1* (*Saa1*) expression and serum SAA1, granulocyte stimulating growth factor (G-CSF), and IL-17 in CCl_4-treated mice with SHED-HepTx. SHED-Heps were treated with siRNA specific for *STC1* (siSTC1) and scrambled control siRNA (siCONT), referred to as siSTC1-SHED-Heps and siCONT-SHED-Heps. CCl_4-treated mice were harvested 4 weeks after transplantation. (**A**) The graphs present the gene expression of *Saa1* in mouse liver by RT-qPCR. The results are presented as a ratio of the expression in the control group (Cont = 1). (**B–D**) The graphs present the serum levels of mouse SAA1 (**B**), G-CSF (**C**), and IL-17 (**D**) by ELISA. **A–D**: Cont, olive oil-treated group; CCl4, CCl_4-treated group; SHED-HepTx, SHED-HepTx group; siCONT, siCONT treatment; siSTC1, siSTC1 treatment. n = 10 in all groups. $^{***}P < 0.005$. ns, no significance. The graph bars represent the mean ± SEM.

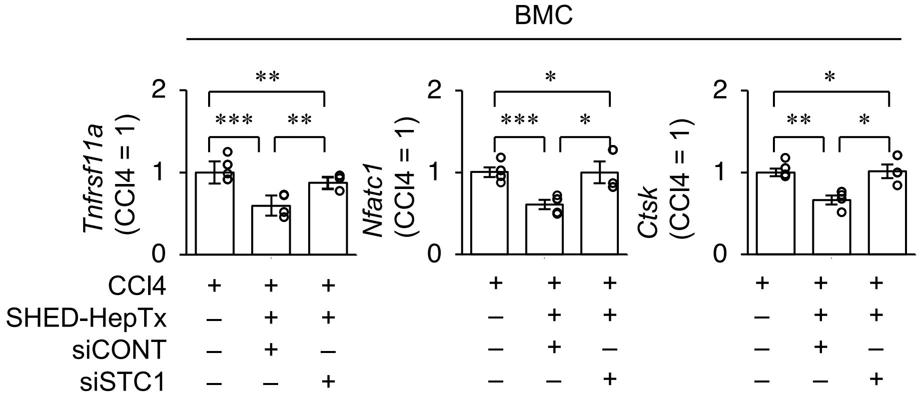

Supplementary Figure 12. Stanniocalcin 1 (STC1) knock-down attenuates the suppression of *in vitro* osteoclast differentiation of BMCs in CCl_4-treated mice with SHED-Tx. SHED-Heps were treated with siSTC1-SHED-Heps and siCONT-SHED-Heps. CCl_4-treated mice were harvested 4 weeks after transplantation. Mouse BMCs were co-cultured with calvarial osteoblasts in the presence of VD_3 (10 nM) and PGE_2 (1 nM). The expression of *Tnfrsf11a*, *Nfatc1*, and *Ctsk* by RT-qPCR. Results are shown as ratios compared to the co-cultures with BMCs derived from CCl_4-treated mice (CCl4 = 1). CCl4, CCl_4-treated group; SHED-HepTx, SHED-HepTx group; siCONT, siCONT treatment; siSTC1, siSTC1 treatment. n = 10 for all groups. $*P < 0.05$, $**P < 0.01$, $***P < 0.005$. The graph bars represent the mean ± SEM.

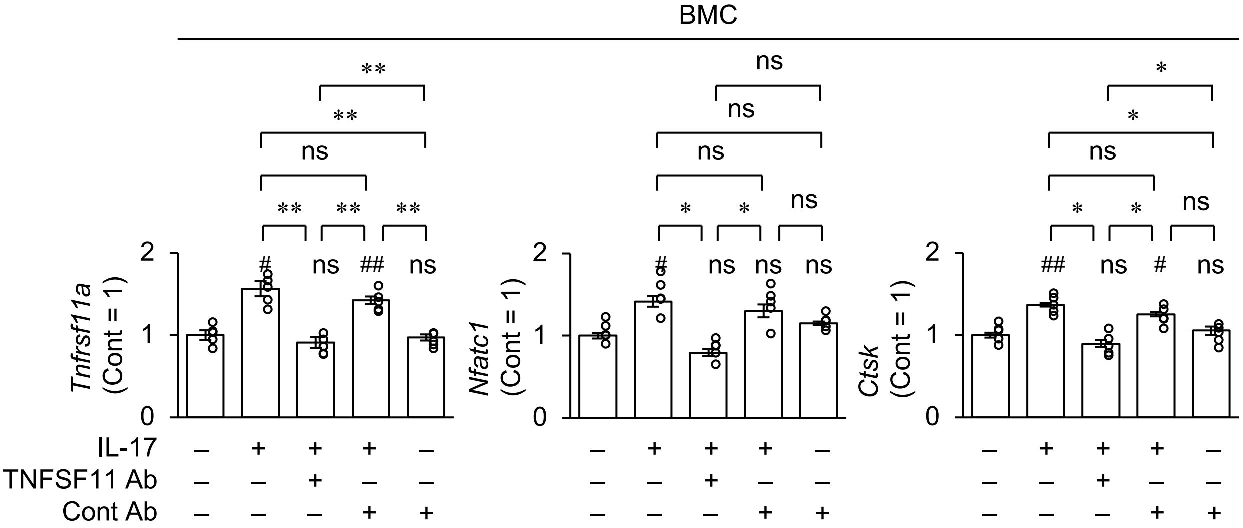

Supplementary Figure 13. Interleukin 17 (IL-17) enhance attenuates the *in vitro* osteoclast differentiation of BMCs via tumor necrosis factor superfamily 11 (TNFSF11). Mouse BMCs were co-cultured with calvarial osteoblasts in the presence of VD_3 (10 nM) and PGE_2 (1 nM). The co-cultures were treated with or without recombinant mouse IL-17 (10 nM) and/or anti-mouse TNFSF11 goat IgG (TNFSF11 Ab; 50 ng/mL) or its control IgG antibody (Cont Ab). The expression of osteoclast markers, including *Tnfrsf11a*, *Nfatc1*, and *Ctsk* by RT-qPCR. Results are shown as ratios compared to the control co- group without IL-17, TNFSF11 Ab, and Cont Ab treatment (Cont = 1). n = 5 for all groups. **P* < 0.05, ***P* < 0.01. ns, no significance. #*P* < 0.05, #*P* < 0.01 vs. control group. ns, no significance vs. control group. The graph bars represent the mean ± SEM.

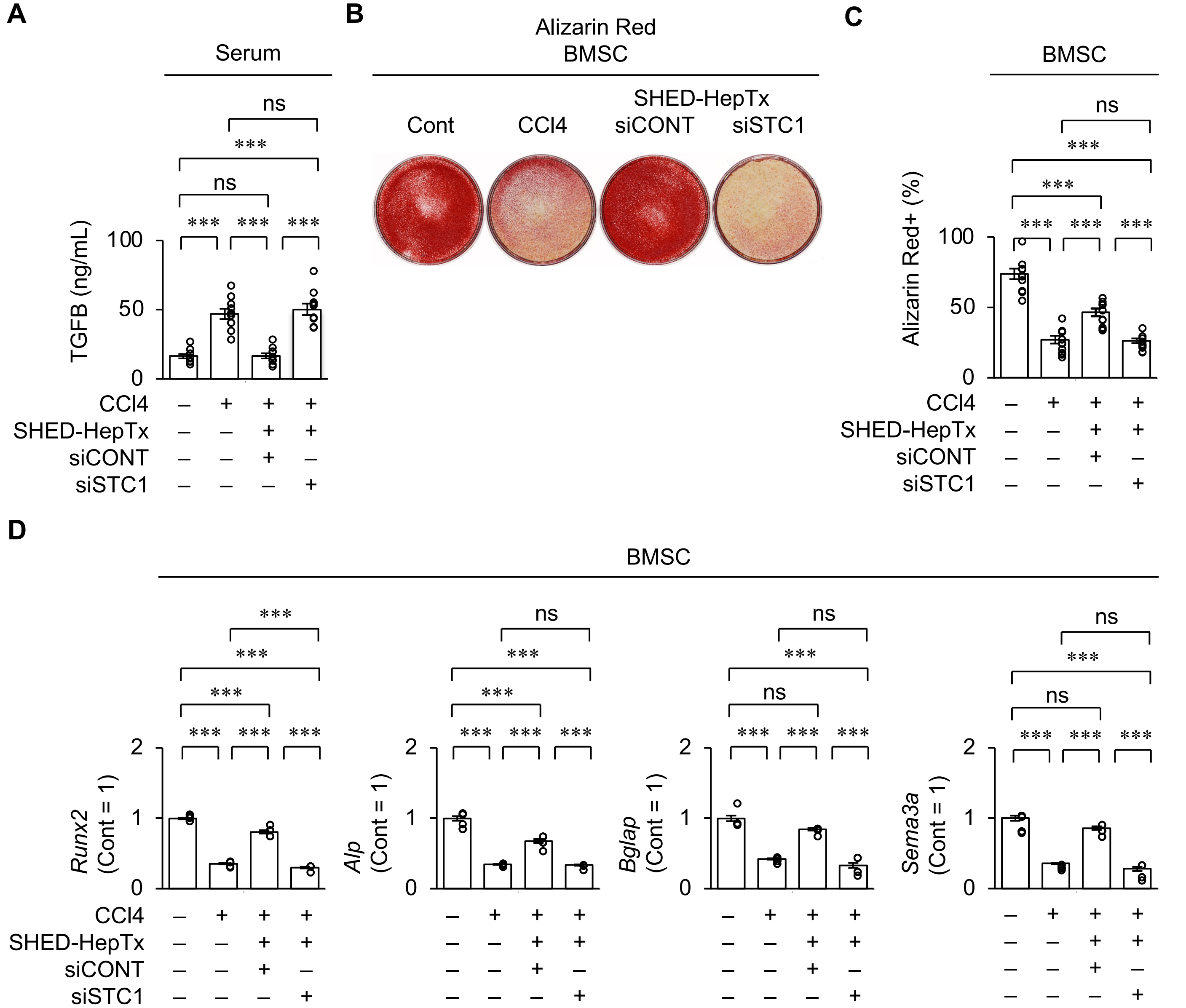

Supplementary Figure 14. STC1 knock-down attenuates the bone formation and expression of semaphoring 3a (Sema3a) in CCl_4-treated mice with SHED-HepTx. (**A**) The graph provides the serum level of transform growth factor beta (TGFB) according to ELISA. (**B–D**) Mouse BMSCs were cultured under an osteogenic induction with AA (50 μg/mL), β-GP (5 mM), and Dex (10 nM). BMSCs were induced for 4 weeks under the osteogenic induction condition. Representative images of mineralized nodule formation were analyzed using Alizarin Red staining (**B**). The graph indicates the ratio of Alizarin Red-positive (Alizarin Red^{+}) area to total area (**C**). BMSCs were induced for 2 weeks under the osteogenic induction condition analyzed by RT-qPCR. The graphs indicate the expression of *Runx2*, *Alp*, *Bglap*, and *Sema3a*, in BMSCs. The results are presented as a ratio to the expression in the control group (Cont = 1) (**D**). **A**–**D**: Cont, olive oil-treated group; CCl4, CCl_4-treated group; SHED-HepTx, SHED-HepTx group; siCONT, siCONT treatment; siSTC1, siSTC1 treatment. **A, C, D**: n = 10 for all groups. $*P < 0.05$, $**P < 0.01$, $***P < 0.005$. ns, no significance. The graph bars represent the mean ± SEM.
